# Unveiling Formation Pathways of Ternary I–III–VI CuInS_2_ Quantum Dots and Their Effect on Photoelectrochemical Hydrogen Generation

**DOI:** 10.1002/advs.202500829

**Published:** 2025-05-28

**Authors:** Hyo Cheol Lee, Hwapyong Kim, Kiwook Kim, Kyunghoon Lee, Wookjin Chung, Seung Beom Ha, Minseo Kim, Eonhyoung Ahn, Shi Li, Seunghyun Ji, Gyudong Lee, Hyeonjong Ma, Sung Jun Lim, Hongsoo Choi, Jae‐Yup Kim, Hyungju Ahn, Su‐Il In, Jiwoong Yang

**Affiliations:** ^1^ Department of Energy Science and Engineering Daegu Gyeongbuk Institute of Science and Technology (DGIST) Daegu 42988 Republic of Korea; ^2^ Department of Chemistry Hong Kong University of Science and Technology (HKUST) Kowloon Hong Kong SAR 999077 Hong Kong; ^3^ Department of Chemical Engineering Dankook University Yongin 16890 Republic of Korea; ^4^ Division of Nanotechnology Daegu Gyeongbuk Institute of Science and Technology (DGIST) Daegu 42988 Republic of Korea; ^5^ DGIST‐ETH Microrobotics Research Center Daegu Gyeongbuk Institute of Science and Technology (DGIST) Daegu 42988 Republic of Korea; ^6^ Department of Robotics and Mechatronics Engineering Daegu Gyeongbuk Institute of science and Technology (DGIST) Daegu 42988 Republic of Korea; ^7^ Department of Chemical Engineering Konkuk University Seoul 05029 Republic of Korea; ^8^ Pohang Accelerator Laboratory Pohang University of Science and Technology (POSTECH) Pohang 37673 Republic of Korea; ^9^ Energy Science and Engineering Research Center Daegu Gyeongbuk Institute of Science and Technology (DGIST) Daegu 42988 Republic of Korea

**Keywords:** I–III–VI, formation mechanism, hydrogen production, photoelectrochemical properties, quantum dots

## Abstract

Understanding the formation mechanisms of semiconductor nanocrystal quantum dots (QDs) is essential for fine‐tuning their optical and electrical properties. Despite their potential in solar energy conversion, the synthesis processes and resulting properties of ternary I–III–VI QDs remain underexplored due to the complex interplay among their constituent elements. Herein, the formation mechanism of ternary I–III–VI CuInS_2_ QDs is investigated, and a direct correlation between their synthesis pathways and photoelectrochemical hydrogen generation performance is established. Two distinct formation pathways governed by the Lewis acid strength of the precursors are revealed. Precursors with weaker Lewis acid strength, such as indium acetate–alkylamine complexes, induce the nucleation of Cu*_x_* S phases, which subsequently transform into CuInS_2_ QDs. Conversely, exemplified by indium iodide–alkylamine complexes, precursors with stronger Lewis acid strength enable the simultaneous incorporation of all elements during nucleation, resulting in the direct formation of CuInS_2_ QDs. Notably, QDs synthesized through this direct pathway exhibit significantly improved electrical properties with lower electron trap densities, resulting in outstanding photoelectrochemical hydrogen production with an excellent photocurrent density of 11.3 mA cm^−2^ at 0.6 V_RHE_ when used as sensitizers in photoanodes. These findings highlight the critical role of formation pathways in tailoring the properties of ternary I–III–VI QDs.

## Graphical abstract

The formation pathways of CuInS_2_ quantum dots are explored through in situ and ex situ characterization. The Lewis acid strength of cation precursors dictates synthesis routes, leading to either indirect or direct nucleation. These routes significantly impact the electrical and photoelectrochemical properties. Notably, CuInS_2_ quantum dots synthesized via direct nucleation exhibit a high photocurrent density of 11.3 mA cm^−2^ for hydrogen production.

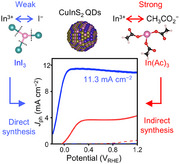

## 1 Introduction

Manipulating the properties of nanomaterials is crucial for fully exploiting their potential across a wide range of applications.^[^ ^1^, ^2^, ^3^, ^4^, ^5^, ^6^, ^7^, ^8^, ^9^, ^10^ ^]^ Tremendous efforts have been directed toward the controlled synthesis of colloidal quantum dots (QDs), driven by their unique size‐ and shape‐dependent properties.^[^ ^1^, ^11^, ^12^, ^13^ ^]^ Significant progress has been achieved with binary semiconductor nanocrystals such as II–VI (CdSe,^[^ ^1^, ^11^ ^]^ CdS^[^ ^1^ ^]^) and IV–VI (PbS,^[^ ^14^, ^15^ ^]^ PbSe^[^ ^15^ ^]^) materials, facilitating precise control of their size and shape, thereby fine‐tuning their optical and electrical properties.^[^ ^12^, ^13^, ^16^, ^17^ ^]^ However, the use of heavy metals (e.g., Cd and Pb) in these QDs raises environmental and health concerns, limiting their practical applications, including energy technologies^[^ ^18^, ^19^ ^]^ and electronic devices.^[^ ^20^, ^21^ ^]^ Consequently, there is a growing demand for developing non‐toxic alternatives to these conventional QDs.^[^ ^22^, ^23^, ^24^ ^]^

Ternary I–III–VI QDs, such as CuInS_2_ (CIS), have emerged as promising alternatives,^[^ ^25^, ^26^, ^27^, ^28^ ^]^ particularly for solar energy applications. They exhibit excellent optical properties, including a large absorption coefficient, tunable bandgap across the visible to near‐infrared (NIR) region, and high photoluminescence (PL) quantum yield.^[^ ^29^, ^30^, ^31^ ^]^ These characteristics make them suitable for diverse applications, such as solar cells,^[^ ^32^, ^33^, ^34^, ^35^ ^]^ photoelectrochemical (PEC) hydrogen generation,^[^ ^36^ ^]^ solar concentrators,^[^ ^37^ ^]^ photodetectors,^[^ ^38^ ^]^ light‐emitting diodes,^[^ ^39^ ^]^ and transistors.^[^ ^40^ ^]^ CIS, a representative material among I–III–VI semiconductors, has been successfully synthesized as nanocrystals with diverse sizes and shapes, including spheres,^[^ ^41^, ^42^, ^43^, ^44^, ^45^ ^]^ platelets,^[^ ^46^, ^47^ ^]^ and hexagons,^[^ ^48^ ^]^ as well as in different crystal phases such as zincblende,^[^ ^49^, ^50^ ^]^ wurtzite,^[^ ^44^, ^45^, ^46^, ^47^, ^48^ ^]^ and chalcopyrite.^[^ ^41^, ^42^, ^43^ ^]^

Understanding the formation mechanism of ternary I–III–VI QDs at the atomic level is essential for optimizing their properties for target applications, because the properties of these QDs are intricately linked to their atomic structure. For instance, the electrical properties of CuInSe_2_ QDs are highly sensitive to the Cu/In ratio, which is attributed to the Cu‐vacancy concentrations.^[^ ^36^ ^]^ Similarly, the concentration of Cu^+^ and Cu^2+^ defects in CIS QDs can be adjusted by varying the Cu/In ratio,^[^ ^51^ ^]^ which also influences their PL characteristics.^[^ ^52^ ^]^ However, investigating the formation mechanisms of these ternary QDs is more challenging than for binary QDs due to the involvement of three constituent elements. Moreover, CIS QDs can exhibit various crystalline phases, including not only the tetragonal chalcopyrite phase^[^ ^41^, ^42^, ^43^ ^]^ but also minor phases such as cubic zincblende^[^ ^49^, ^50^ ^]^ and hexagonal wurtzite.^[^ ^44^, ^45^, ^46^, ^47^, ^48^ ^]^ The potential for diverse reaction pathways further adds to the complexity of these systems. For example, previous studies have demonstrated the transformation of Cu*_x_* S nanocrystals via cation exchange, resulting in cubic^[^ ^50^ ^]^ or hexagonal phases^[^ ^44^, ^45^, ^46^, ^47^, ^48^ ^]^ depending on reaction conditions.

Herein, we elucidate the formation mechanism of CIS QDs with the tetragonal chalcopyrite structure and investigate how synthesis pathways affect their PEC hydrogen generation performance. Using a combination of in situ and ex situ characterization methods, we demonstrate that the Lewis acid strength of the cation precursors governs the formation pathways. Specifically, when indium acetate–oleylamine (In(Ac)_3_–OAm) complexes are used as precursors, the Cu*_x_* S intermediates form initially, followed by their transformation into CIS QDs during the growth stage. In contrast, using indium iodide–oleylamine (InI_3_–OAm) complexes leads to the direct nucleation of CIS QDs from the onset of the QD formation. Notably, this difference in formation pathways significantly affects the electrical and PEC properties of these two‐types of QDs, despite their similar morphologies and optical characteristics. Photoanodes employing CIS QDs synthesized via the direct synthesis route achieve enhanced PEC hydrogen production with a remarkable photocurrent density of 11.3 mA cm^−2^ at 0.6 V_RHE_. This exceptional performance is achieved without requiring additional steps such as shelling, alloying, or doping, suggesting the critical role of formation pathways in determining the properties of ternary QDs.

## 2 Results and Discussion

**Figure 1:**
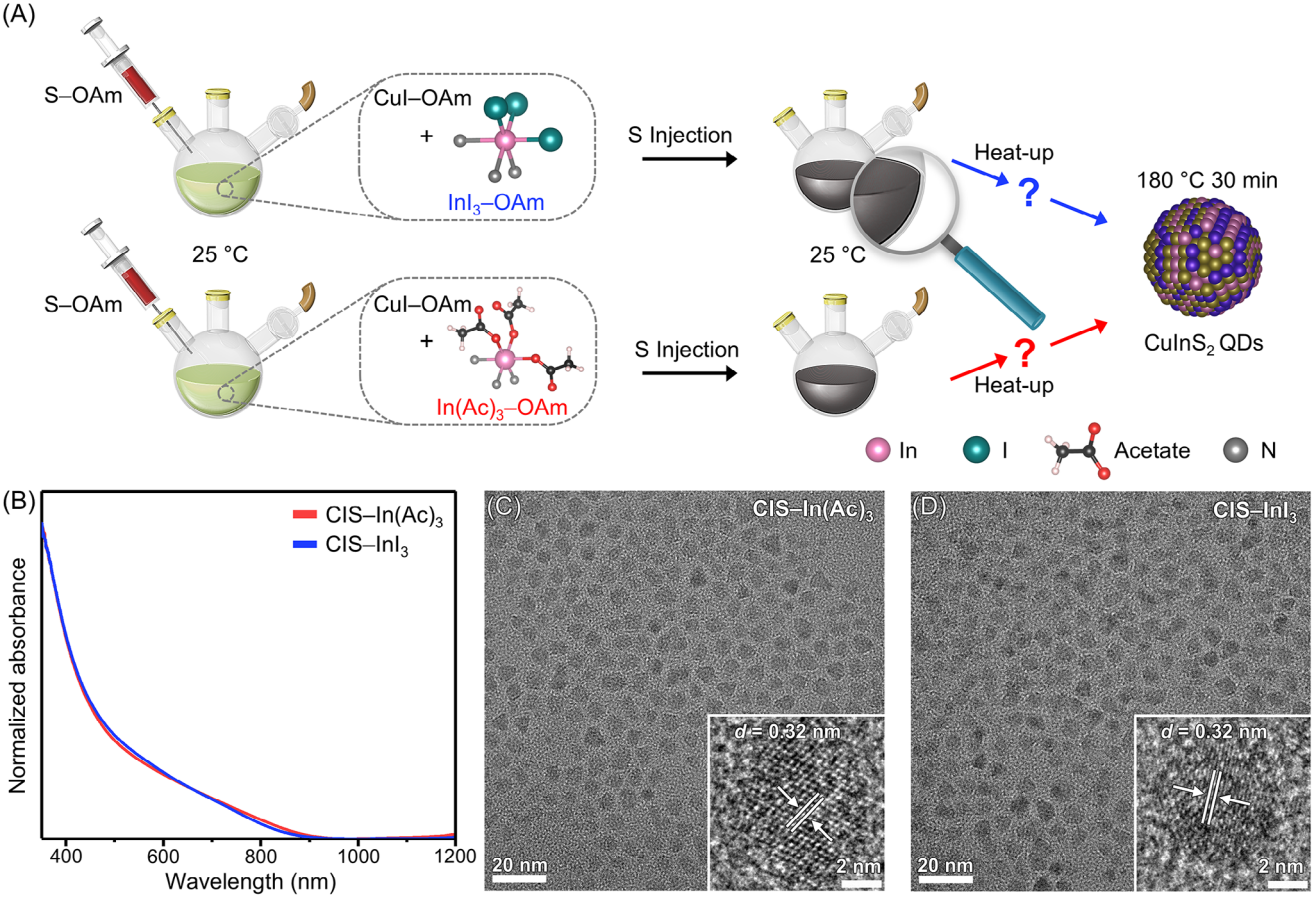
Synthesis of CIS QDs. A) Schematic illustration depicting two distinct synthesis routes of CIS QDs. Note that the illustrations should be interpreted as conceptual representations of molecular species, not precise depictions of specific structural geometries (e.g., bond lengths, angles between atoms, and ionic radii). B) Absorption spectra of two‐types of CIS QDs. TEM images of CIS QDs synthesized using C) In(Ac)_3_–OAm and D) InI_3_–OAm complexes. Each inset shows a high‐resolution TEM image.

CIS QDs were selected as model systems to investigate the formation mechanism of I–III–VI QDs. These QDs were synthesized through a heat‐up method employing Lewis acid–base reaction between metal–oleylamine (OAm) and S–OAm complexes (**Figure** **1**, see Experimental Section for experimental details).^[^ ^35^, ^53^ ^]^ Copper(I) iodide–OAm complexes were employed as Cu precursors, while either InI_3_–OAm or In(Ac)_3_–OAm complexes were used as In precursors (Figure 1A). It has been known that In(III) species typically bind with three OAm molecules to form complexes.^[^ ^54^ ^]^ All other synthesis conditions remained consistent across experiments. CIS QDs synthesized employing In(Ac)_3_–OAm complexes are referred to as CIS–In(Ac)_3_ QDs, and those synthesized with InI_3_–OAm complexes are termed CIS–InI_3_ QDs.

Both CIS–In(Ac)_3_ and CIS–InI_3_ QDs exhibit similar absorption profiles in the visible and NIR regions (Figure 1B) and have comparable bandgaps of ≈1.6 eV (Figure S1A, Supporting Information), indicating weak quantum confinement effects (≈bulk bandgap: ≈1.4–1.5 eV).^[^ ^55^ ^]^ Notably, the PL from CIS QDs originates not from their band‐edge transition but from donor–acceptor pair emissions, primarily associated with Cu defect‐induced PL (Figure S1B, Supporting Information).^[^ ^51^, ^55^ ^]^ The atomic compositions of both types of CIS QDs are also similar, being In‐rich (Table S1, Supporting Information), which is common in I–III–VI QDs.^[^ ^36^, ^47^, ^49^, ^51^ ^]^ X‐ray diffraction (XRD) and transmission electron microscopy (TEM) analyses indicate that both types of CIS QDs possess the chalcopyrite phase crystal structures (Figure 1C,D and Figure S2, Supporting Information). A reflection near 32°, which is characteristic of the cubic phase,^[^ ^49^, ^50^ ^]^ is not apparent, confirming that our CIS QDs are in the tetragonal phase.^[^ ^35^ ^]^ The estimated particle sizes of CIS–In(Ac)_3_ QDs and CIS–InI_3_ QDs are ≈7.6 and ≈7.2 nm, respectively, falling within the error range (Figure S3, Supporting Information). Because OAm serves as the sole ligand and solvent for the QD synthesis, both CIS QDs are preliminarily passivated by OAm (Figure S4, Supporting Information), and these OAm‐passivated QDs were utilized throughout this study unless otherwise specified.^[^ ^38^ ^]^

**Figure 2:**
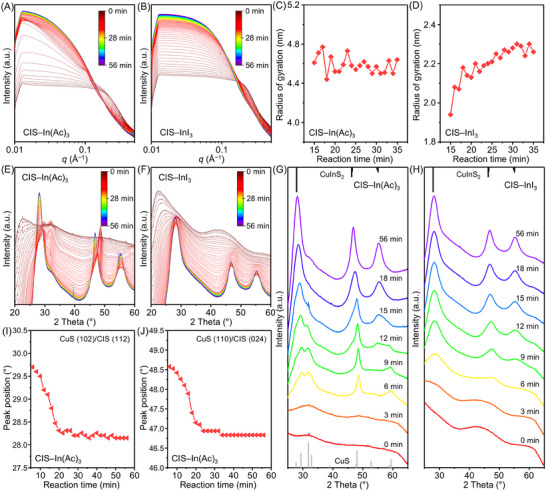
In situ X‐ray scattering analysis on the formation of CIS QDs. In situ SAXS patterns depicting the evolution of A) CIS–In(Ac)_3_ and B) CIS–InI_3_ QDs. The scattering intensities of panels A and B are plotted using a logarithmic scale. Estimated radius of gyration of intermediates during the synthesis of C) CIS–In(Ac)_3_ and D) CIS–InI_3_ QDs. In situ WAXS patterns depicting the evolution of E) CIS–In(Ac)_3_ and F) CIS–InI_3_ QDs as a function of reaction time. The representative scattering patterns acquired from G) panel E and H) panel F. Reflections of bulk CuS (JCPDS No. 79–2321) and CuInS_2_ (JCPDS No. 47–1372) are presented as gray and black bars, respectively. The scattering intensities of panels E–H are plotted using a linear scale. Peak position shifts of I) CuS (102)/CIS (112) and J) CuS (110)/CIS (024) planes as a function of reaction time (corresponding to 6–55 min). In this work, the first recorded in situ SAXS and WAXS patterns at room temperature are denoted as 0 min.

To elucidate the formation process of CIS QDs, we conducted in situ simultaneous small‐angle X‐ray scattering (SAXS) and wide‐angle X‐ray scattering (WAXS) measurements (**Figure** **2**, see Experimental Section for experimental details). The SAXS profiles for the entire reaction periods of the synthesis of two‐types of QDs are presented in Figure 2A,B, respectively. During the initial synthesis stage of CIS–In(Ac)_3_ QDs, transient reflections were observed, which diminished at 10 min (Figure 2A; Figure S5A, Supporting Information). Meanwhile, no distinct reflections were detected for the synthesis of CIS–InI_3_ QDs (Figure 2B; Figure S5B, Supporting Information). An additional experiment was conducted to track the exact positions of the peaks, involving annealing the reaction solution at 40 °C for 10 min (Figure S6, Supporting Information). The positions of the first‐ and second‐order reflections are at *q*_1_ = 0.11 Å^−1^ (*d* = 5.71 nm) and *q*_2_ = 0.22 Å^−1^, respectively (*q*_1_:*q*_2_ = 1:2), suggesting that they originate from periodic assemblies of the lamellar structures.^[^ ^42^, ^43^ ^]^ SAXS measurements on various precursor complexes confirm that these lamellar structures do not arise from the precursor complexes (Figure S7, Supporting Information). A more detailed discussion on the nature of these lamellar intermediates is provided later in this paper.

To gain a better understanding of two different formation pathways, quantitative analysis on the radii of gyration during the initial growth stage was performed (Figure 2C,D; Table S2, Supporting Information). It is important to note that the radii of gyration are often larger than the particle size estimated by TEM, particularly for nanoparticles smaller than 10 nm.^[^ ^56^, ^57^ ^]^ Interestingly, the radius of gyration for CIS–InI_3_ QDs gradually increased during the growth stage, while that for CIS–In(Ac)_3_ QDs showed no clear trend and was considerably large from the beginning of synthesis. TEM analyses of intermediate nanocrystals during the synthesis support these findings (Figures S8 and S9, Supporting Information). The nucleation of relatively large and polydisperse particles, including triangular‐like 2D morphologies, at the onset of CIS–In(Ac)_3_ QD synthesis is attributed to the growth pathway involving the lamellar intermediates.^[^ ^58^, ^59^, ^60^ ^]^

WAXS analysis provides information on the crystal structure evolution during the QD formation. Initially, in the first 0−3 min of the synthesis, WAXS patterns for both types of QDs showed no distinct reflection peaks (Figure 2E–H), indicating a lack of crystallinity. During the synthesis of CIS–In(Ac)_3_ QDs, starting at ≈6 min, a series of reflection patterns emerged at 29.7, 31.9, 48.6, and 59.3° (Figure 2G). This period signifies a critical stage where lamellar reflections become most pronounced, as observed in the SAXS analysis. The positions of these reflections match those of (102), (103), (110), and (116) planes of bulk hexagonal CuS, respectively, suggesting the formation of Cu*_x_* S intermediates. Notably, the reaction time at which the Cu*_x_* S phase signals were most pronounced in WAXS coincides with the time when lamellar patterns were most prominent in SAXS. This correlation strongly suggests that the lamellar structures originate from assemblies of OAm‐passivated Cu*_x_* S intermediates.

In 9−18 min, after lamellar patterns vanished, the (102) and (110) plane peaks gradually shifted to lower angles (Figure S10, Supporting Information). By the end of the reaction, these peak positions shifted from 29.7° to 28.1° and from 48.6° to 46.8° (Figure 2I,J), aligning with the (112) and (024) planes of bulk tetragonal CIS. The emergence of an additional reflection near 55.3°, corresponding to the (116)/(132) planes of CIS, was also observed. The coexistence of reflections from CuS and CIS phases and the gradual shift from CuS to CIS peaks supports the transformation of Cu*_x_* S into CIS–In(Ac)_3_ QDs rather than the complete dissolution of Cu*_x_* S phases followed by the subsequent nucleation of CIS QDs. The weak retention of Cu_x_S signals at the end of the SAXS experiment (Figure 2G) is likely due to the in situ X‐ray scattering setup, in which the reaction mixture was loaded into a capillary without stirring, making it challenging to ensure complete reaction. In contrast, the results on the synthesis of CIS–InI_3_ QDs show reflections from tetragonal CIS crystal phases from the onset (Figure 2H), indicating that CIS–InI_3_ QDs form without intermediate phases. Ex situ XRD analysis further supports this finding, showing the clear disappearance of the Cu*_x_* S peak near 31.9° (Figure S11, Supporting Information).

**Figure 3:**
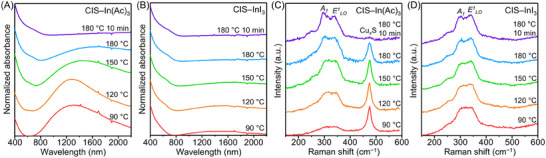
Ex situ absorption and Raman spectroscopy of the formation of CIS QDs. Temporal evolution of absorption spectra during the synthesis of A) CIS–In(Ac)_3_ and B) CIS–InI_3_ QDs. Temporal evolution of Raman spectra during the synthesis of C) CIS–In(Ac)_3_ and D) CIS–InI_3_ QDs. For ex situ measurements, reaction stages are indicated by temperature and reaction time. For example, the sample labeled “180 °C 10 min” indicates that the synthesis was carried out for 10 min at 180 °C. Samples without a time component (90, 120, 150, and 180 °C) represent the temperatures at which their synthesis was halted during the heating step. This naming convention is used consistently throughout the rest of the paper for ex situ measurements.

The temporal changes in the absorption spectra during the synthesis of CIS–In(Ac)_3_ QDs are displayed in **Figure** **3**A. At 90 °C, the spectrum showed a broad absorption profile extending through the NIR region (800–2200 nm), attributed to the localized surface plasmon resonances of Cu*_x_* S nanocrystals.^[^ ^61^ ^]^ This implies the formation of intermediate Cu*_x_* S nanocrystals, consistent with the results of WAXS analysis. The absorption of the NIR band decreased with the increasing temperature, accompanied by a peak shift toward longer wavelengths. This shift suggests an increasing dominance of Cu^+^ over Cu^2+^ in the nanocrystals,^[^ ^61^ ^]^ indicating the transformation of Cu*_x_* S into CIS.^[^ ^44^, ^45^, ^46^, ^47^, ^48^, ^62^ ^]^ After 10 min of reaction at 180 °C, absorption from the CIS was predominant. Subsequently, the morphology of the particles gradually evolved into a spherical shape as they grew (Figure 1C and Figure S8, Supporting Information). In contrast, during the synthesis of CIS–InI_3_ QDs (Figure 3B), the absence of the plasmon resonance in the absorption spectra suggests the direct formation of CIS QDs without the formation of the Cu*_x_* S phase, which aligns with the WAXS data. The experimental results remain consistent even at elevated temperatures of 210 °C (Figure S12, Supporting Information), suggesting that the proposed reaction pathways do not change with increasing temperature.

Raman spectroscopy provides further insights into the reaction dynamics (Figure 3C). Two distinct vibrational modes at 294 and 341 cm^−1^ can be assigned to *A_1_* and *E^1^_LO_* modes of the chalcopyrite CIS phase, respectively,^[^ ^63^ ^]^ and a sharp peak at 474 cm^−1^ is characteristic of the Cu*_x_* S phase.^[^ ^64^ ^]^ During the synthesis of CIS–In(Ac)_3_ QDs, the intensity of the Cu*_x_* S peak diminished, while those from CIS became more pronounced. This change, accompanied by a broadening of the Cu*_x_* S peak linewidth, supports the suggested transformation mechanism. In contrast, Raman spectra of CIS–InI_3_ QDs do not show the Cu*_x_* S peak (Figure 3D). This is consistent with the WAXS and absorption data, indicating that CIS QDs crystallize directly without forming Cu*_x_* S intermediates.

**Figure 4:**
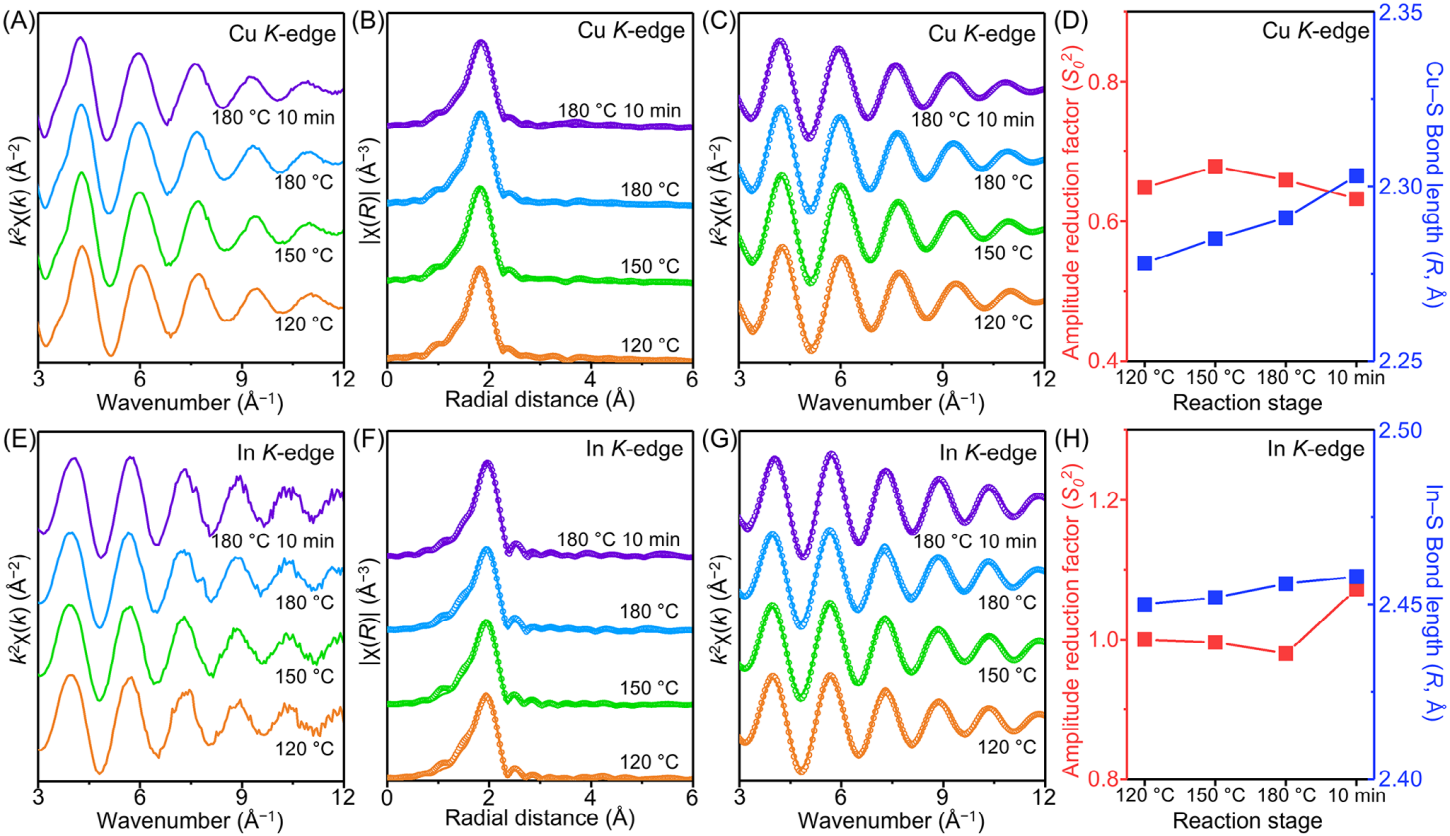
X‐ray absorption analysis of the formation of CIS–In(Ac)_3_ QDs. Cu *K*‐edge A) experimental *k* ^2^‐weighted EXAFS oscillations, B) Fourier‐transformed EXAFS spectra, and C) Fourier‐filtered EXAFS spectra of intermediates during the synthesis of CIS–In(Ac)_3_ QDs. D) Cu─S amplitude reduction factor (*S_0_* ^2^) and bond length (*R*, Å), estimated by EXAFS fitting analysis. In *K*‐edge E) experimental *k* ^2^‐weighted EXAFS oscillations, F) Fourier‐transformed EXAFS spectra, and G) Fourier‐filtered EXAFS spectra of intermediates during the synthesis of CIS–In(Ac)_3_ QDs. H) In─S amplitude reduction factor (*S_0_* ^2^) and bond length (*R*, Å), estimated by EXAFS fitting analysis. Solid lines represent fitted data, while circled patterns represent experimental data.

To investigate the local environment around each cation during the formation of CIS–In(Ac)_3_ QDs, we conducted extended X‐ray absorption fine structure (EXAFS) analysis (**Figure** **4**). The experimental and fitted curves for both Cu (Figure 4A–D) and In (Figure 4E–H) in all samples exhibit good agreement (Tables S3 and S4, Supporting Information). The Cu─S amplitude reduction factors (*S_0_* ^2^) remained almost constant throughout the reaction (Figure 4D), implying no significant change in the coordination number around Cu atoms. However, the Cu─S bond length showed a slight increase, from 2.278 Å (at 120 °C) to 2.303 Å (after 10 min at 180 °C). This increase in bond length is attributed to the incorporation of In^3+^ ions, which have a larger effective ionic radius (0.80 Å) compared to Cu^2+^ (0.73 Å) or Cu^+^ (0.77 Å) ions.^[^ ^65^ ^]^ Conversely, in the case of CIS–InI_3_ QDs, the Cu─S bond length exhibited no significant change throughout the reaction (Figure S13 and Table S5, Supporting Information). In addition, the In─S amplitude reduction factors for CIS–In(Ac)_3_ QDs remained constant until 180 °C, and a slight increase was noted in the sample annealed at this temperature for 10 min (Figure 4H). This suggests the integration of surface‐located In ions into the internal crystal domains. Overall, these EXAFS analysis results support the hypothesis that the synthesis of CIS–In(Ac)_3_ QDs involves the initial formation of Cu*_x_* S phases, which then transition to CIS through the incorporation of In ions.

**Figure 5:**
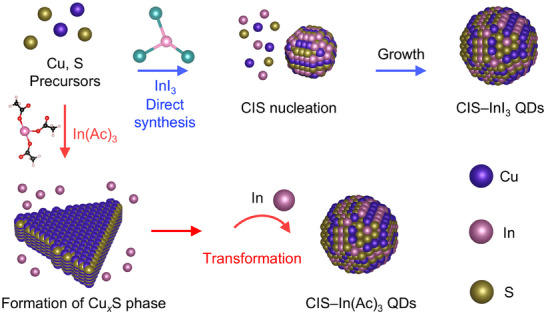
Schematic illustration depicting the formation of CIS QDs with two different indium precursors. Red and blue arrows indicate synthesis pathways of CIS–In(Ac)_3_ and CIS–InI_3_ QDs, respectively.

We propose the formation mechanisms for two distinct types of CIS QDs (**Figure** **5**). For the QDs synthesized with In(Ac)_3_–OAm complexes, the process begins with the formation of polydisperse Cu*_x_* S nanoparticles. The next stage involves the transformation of Cu*_x_* S into CIS phases, accompanied by their growth into sphere‐like shapes. In contrast, the synthesis of CIS–InI_3_ QDs follows a different path that does not involve the transformation process between Cu*_x_* S and CIS crystals. Instead, this process is characterized by the simultaneous involvement of all constituent elements in small CIS QDs right from nucleation, followed by gradual growth into larger QDs. The differences in the formation pathways of CIS QDs can be interpreted as the result of Lewis acid‐base reactions involving metal–alkylamine and S–alkylamine complexes. The hard‐soft acid‐base theory offers insights into the Lewis acid strengths of In precursors. Notably, the hard acid In^3+^ ions form stronger bonds with the hard base RCOO^−^ (in In(Ac)_3_–OAm complexes) than with the soft base I^−^.^[^ ^66^, ^67^ ^]^ This stronger bonding reduces the Lewis acid strength in In(Ac)_3_–OAm complexes,^[^ ^67^ ^]^ leading to the initial formation of the lamellar structure and Cu*_x_* S phase. Triangular‐like polydisperse shapes of Cu*_x_* S intermediate nanoparticles can be attributed to the formation of lamellar structures. In contrast, the weaker interaction between In^3+^ and the soft base I^−^ (in InI_3_–OAm complexes) allows more facile release of In^3+^ ions, promoting the direct nucleation of CIS QDs.

**Figure 6:**
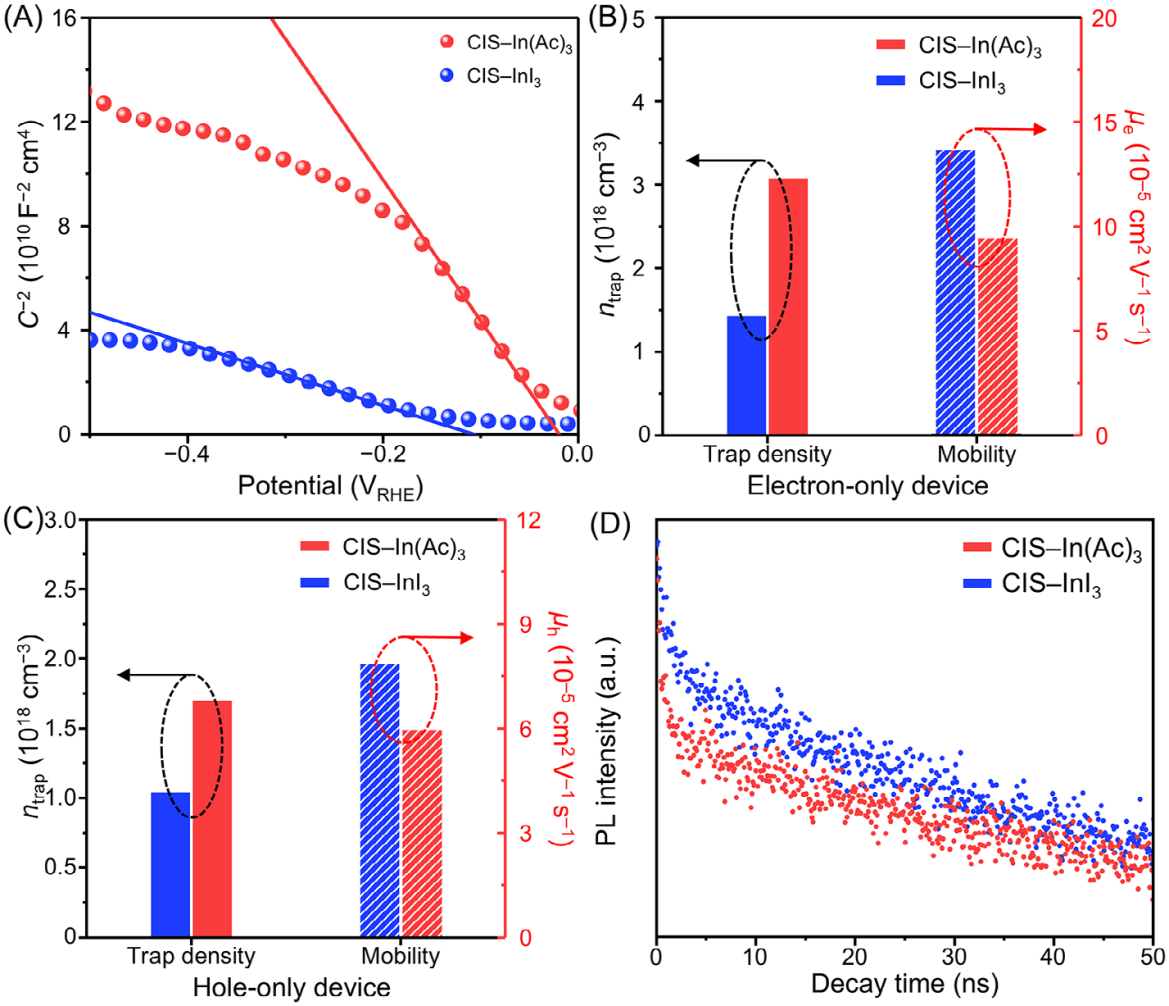
Optical and electrical characteristics of CIS QDs. A) Mott–Schottky plot for CIS QDs. Trap density and carrier mobility for B) electron‐only and C) hole‐only devices employing CIS QDs, as calculated from the SCLC plots. D) Time‐resolved photoluminescence curves of CIS−In(Ac)_3_ and CIS−InI_3_ QDs. The PL intensity is plotted using a linear scale. Pristine CIS QDs with OAm ligands were utilized for Mott–Schottky and SCLC analyses, without any post‐synthetic ligand treatments.

We investigated the influence of different formation pathways on the electrical properties of QDs (**Figure** **6**). Mott–Schottky plots (Figure 6A, see Methods Section, Supporting Information, for experimental details) reveal that both types of CIS QDs exhibit typical *p*‐type characteristics, indicated by their negative slopes. The measured acceptor concentration (*N*_a_) of CIS–InI_3_ QDs (1.17 × 10^20^ cm^−3^) is much higher than that of CIS–In(Ac)_3_ QDs (2.57 × 10^19^ cm^−3^). Additionally, we estimated the trap density (*n*_trap_) and carrier mobility (*µ*) using the space‐charge‐limited‐current (SCLC) method (see Methods Section, Supporting Information, for experimental details), showing that CIS–InI_3_ QDs exhibit reduced trap densities and enhanced carrier mobility compared to CIS–In(Ac)_3_ QDs (Figure 6B,C, Figures S14 and S15, and Table S6, Supporting Information). These findings are consistent with the longer carrier lifetime observed for CIS–InI_3_ QDs compared to CIS–In(Ac)_3_ QDs. (Figure 6D). The increase in trap densities in CIS–In(Ac)_3_ QDs is attributed to the rearrangement of S atoms during the synthesis. In the Cu*_x_* S phase, S atoms are organized in a close‐packed arrangement (hexagonal or cubic).^[^ ^67^ ^]^ However, this arrangement is likely to deviate during the transformation into CIS QDs, owing to the differences in bond lengths between Cu─S and In─S.^[^ ^29^ ^]^ This observation is consistent with the EXAFS analysis, showing the increase in the Cu─S bond length (Figure 4D). It has been suggested that conditions facilitating the easy migration of atoms can induce defect formation.^[^ ^68^, ^69^ ^]^

**Figure 7:**
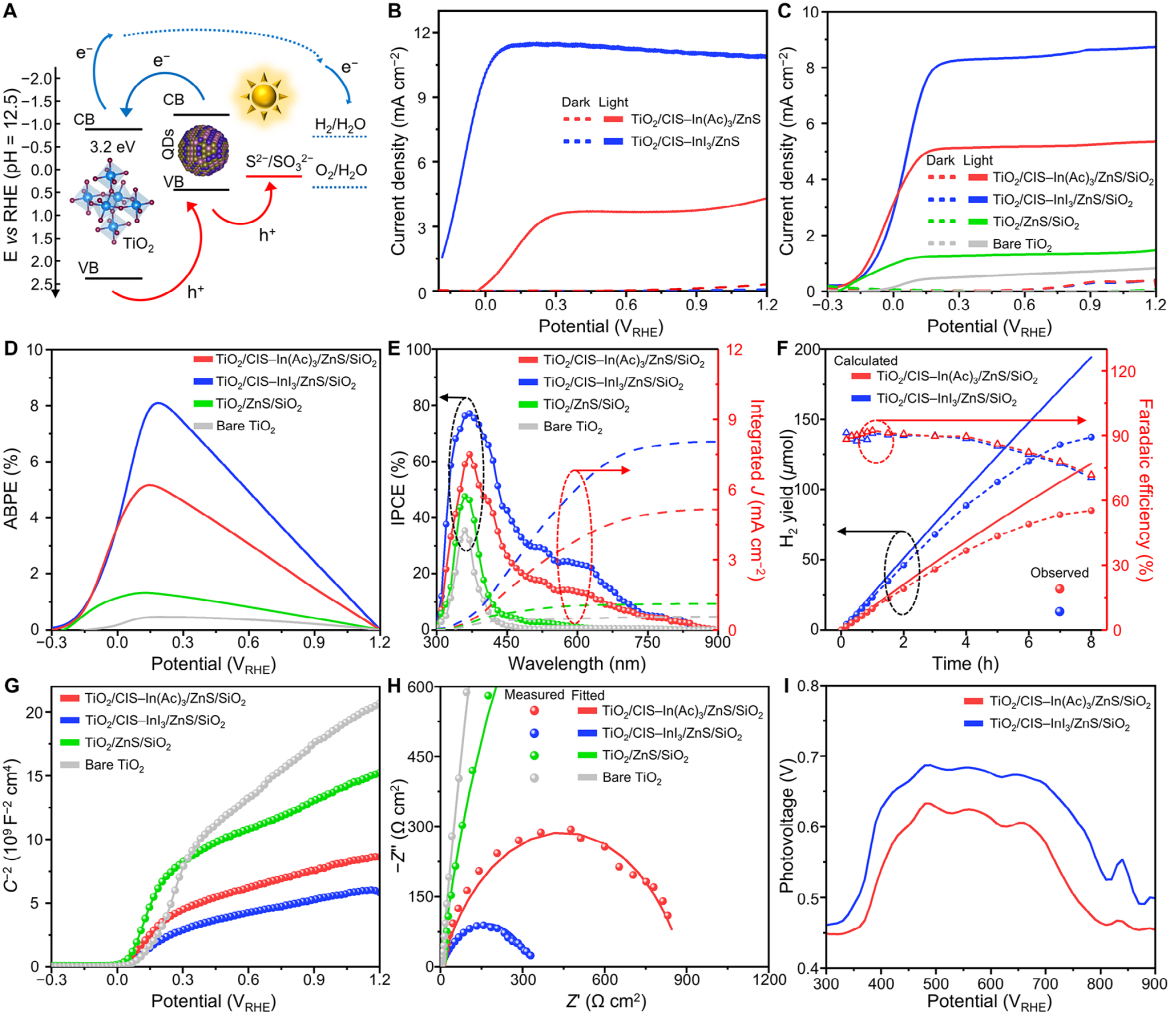
PEC characteristics of photoanodes utilizing CIS QDs. A) Schematic illustration depicting the energy levels and charge transfer mechanism in PEC devices using CIS QD‐sensitized TiO_2_ photoanodes. Current–voltage (*J*–*V*) curves of B) the TiO_2_/CIS QDs/ZnS and C) the TiO_2_/CIS QDs/ZnS/SiO_2_ photoanodes. D) ABPE curves of CIS‐QDs sensitized TiO_2_ photoanodes measured at 0.6 V_RHE_ applied potential under AM 1.5G illumination. E) IPCE spectra and integrated photocurrent density of the TiO_2_/CIS QDs/ZnS/SiO_2_ photoanodes measured at 0.6 V_RHE_ applied potential under AM 1.5G illumination. F) PEC hydrogen evolution amount experimentally measured and calculated, and faradaic efficiency plots of the TiO_2_/CIS QDs/ZnS/SiO_2_ photoanodes as a function of time measured at 0.6 V_RHE_ applied potential under AM 1.5G illumination. G) Mott–Schottky plots of the photoanodes. H) Nyquist plots of the photoanodes under AM 1.5G illumination. I) Surface photovoltages of CIS‐QDs sensitized TiO_2_ photoanodes.

Finally, we examined the PEC characteristics of CIS QD‐sensitized TiO_2_ photoanodes^[^ ^70^, ^71^, ^72^, ^73^, ^74^ ^]^ (Figure S16, Supporting Information, see Methods Section, Supporting Information for experimental details), using a quartz‐type cell with 0.25 m Na_2_S and 0.35 m Na_2_SO_3_ (pH 12.5). The charge transfer process in CIS QD‐sensitized TiO_2_ photoanode is depicted in **Figure** **7**A. Photogenerated electrons from CIS QDs and TiO_2_ move to the counter electrode via the electrical circuit for the hydrogen evolution reaction. Simultaneously, photogenerated holes from TiO_2_ and CIS QDs contribute to the redox reaction of the electrolyte. The photocurrent densities of TiO_2_ photoanodes sensitized with CIS–InI_3_ QDs and CIS–In(Ac)_3_ QDs, both coated with ZnS passivation layers (three monolayers, ≈0.8–1.0 nm),^[^ ^75^ ^]^ are 11.3 and 3.7 mA cm^−2^ at 0.6 V_RHE_, respectively (Figure 7B). Notably, the obtained photocurrent density using CIS–InI_3_ QDs is superior to previously reported values for CIS^[^ ^76^, ^77^ ^]^ and CuInSe_2_^[^ ^36^, ^78^, ^79^ ^]^ QD‐based PEC hydrogen generation devices (Table S7, Supporting Information). This improvement is remarkable since it is achieved solely through controlling the synthesis process of pure CIS QDs, without resorting to complicated processes such as shelling, alloying, or doping with other elements.^[^ ^76^, ^77^, ^78^, ^79^, ^80^, ^81^, ^82^, ^83^, ^84^ ^]^

To increase the stability of the QD‐sensitized photoanodes, additional SiO_2_ passivation layers (≈0.8 nm)^[^ ^34^, ^36^ ^]^ were coated for the subsequent PEC experiments (Figure 7C). The resulting double passivated photoanodes demonstrate photocurrent densities of 8.3 and 5.1 mA cm^−2^ for CIS–InI_3_ and CIS–In(Ac)_3_ QDs (at 0.6 V_RHE_), respectively (≈1.4 mA cm^−2^ for TiO_2_/ZnS/SiO_2_ photoanodes without QDs). Furthermore, the applied bias photon‐to‐current conversion efficiency (ABPE, Figure 7D) and incident photon‐to‐current efficiency (IPCE, Figure 7E) of TiO_2_/CIS–InI_3_ QDs/ZnS/SiO_2_ photoanodes are significantly higher than those of TiO_2_/CIS–In(Ac)_3_ QDs/ZnS/SiO_2_ photoanodes. Notably, both electrodes exhibit almost identical absorption spectra (Figure S17, Supporting Information), suggesting that the difference in PEC performances of CIS QD‐sensitized photoanodes is attributed to the distinct electrochemical characteristics of the QDs, rather than other factors such as light absorption or QD loading amounts.

The amount of the actual hydrogen evolved was measured, and the photoanodes using CIS–InI_3_ and CIS–In(Ac)_3_ QDs produced 137.2 and 85.0 µmol of hydrogen over 8 h, respectively, with both maintaining Faradaic efficiencies of ≈70% (Figure 7F). Chronoamperometry analysis shows that the photoanodes exhibit stability comparable to that of recent studies using heavy‐metal‐free QD‐sensitized photoanodes,^[^ ^36^, ^81^, ^84^ ^]^ although further improvements are required to match those using Cd‐based QD‐sensitizers (Figure S18, Supporting Information).^[^ ^82^ ^]^ Additionally, the CIS QD‐sensitized photoanodes exhibit stable photocurrent densities during cycle tests (Figure S19, Supporting Information). We examined the chemical and structural changes in photoanodes after 10 h of PEC hydrogen generation (Figures S20–S25, Supporting Information) using XRD, scanning electron microscopy (SEM), and X‐ray photoelectron spectroscopy (XPS) analyses. The position of the (112) peak of CIS in the XRD pattern remained constant, indicating that the crystal structures of the QDs were preserved (Figure S20, Supporting Information). SEM analysis showed no significant structure changes, including the elemental distribution, suggesting that the photoanodes maintained their structural integrity (Figures S21–S24, Supporting Information). However, all XPS peaks shifted to higher binding energies with increasing operation time (Figure S25, Supporting Information), indicating that partial surface oxidation of the CIS QDs and ZnS layers is a major factor in the degradation of the photoanodes.

To further understand the electrochemical characteristics of the photoanodes employing QDs, Mott–Schottky, electrochemical impedance spectroscopy (EIS), and electron lifetime analyses were conducted (Figure 7G–I). Photoanodes using CIS–InI_3_ QDs exhibit higher donor concentrations (*N*_d_) and more negative flat band potential (*V*_FB_) than the photoanodes using CIS–In(Ac)_3_ QDs (Figure 7G and Table S8, Supporting Information), suggesting more efficient electron transport due to enhanced charge separation. EIS analysis confirms a lower charge transfer resistance between the electrolyte interfaces and photoanodes employing CIS–InI_3_ QDs (Figure 7H; Table S9, Supporting Information). This reduced charge transfer resistance is attributed to minimized nonradiative and interfacial charge recombination in the TiO_2_/CIS–InI_3_ QDs/ZnS/SiO_2_ photoanodes. Consequently, a longer electron lifetime is observed in surface photovoltage measurements (Figure 7I). These results are consistent with the diminished open‐circuit voltage (V_oc_) (Figure S26, Supporting Information) and the prolonged electron lifetime of TiO_2_/CIS–InI_3_ QDs/ZnS/SiO_2_ photoanodes, as estimated from open‐circuit voltage decay (Figure S27, Supporting Information). Overall, these findings suggest that the difference in the synthesis pathways of QDs can govern their final properties.

To explore the applicability of CIS QDs beyond TiO_2_ photoanodes, we prepared BiVO_4_ photoanodes sensitized with both types of CIS QDs (See Methods Section, Supporting Information for experimental details). Notably, the photocurrent density of BiVO_4_ photoanodes with CIS–InI_3_ QDs is higher compared to those with CIS–In(Ac)_3_ QDs (Figure S28, Supporting Information), suggesting that our findings are broadly applicable, regardless of the choice of photoanode materials.

## 3 Conclusion

In summary, this study has elucidated the formation mechanism of ternary I–III–VI CIS QDs and their subsequent impact on the electrical properties of the resulting QDs, as well as their PEC properties when used as sensitizers in photoanodes. A key determinant in these formation pathways is the difference in Lewis acid strength within the cation precursors. Specifically, when In(Ac)_3_–OAm complexes serve as the indium precursor, the strong interaction between In^3+^ and RCOO^−^ limits the Lewis acid strength of these complexes, thereby resulting in the nucleation of the Cu*_x_* S phase. Subsequently, In ions are incorporated into the Cu*_x_* S phase during the growth stage, transforming it into CIS QDs. In contrast, employing InI_3_–OAm complexes, which fosters a weaker interaction between In^3+^ and I^−^, leads to the direct nucleation and subsequent growth of chalcopyrite CIS QDs. Despite similarities in their morphologies, optical bandgaps, and compositions, QDs synthesized via these two distinct pathways exhibit different electrical and PEC properties. Notably, the photoanodes utilizing CIS QDs formed through the direct approach exhibit enhanced electrical and PEC performances, primarily due to reduced trap densities. This research provides crucial insights into the fundamental synthesis mechanism of ternary I–III–VI QDs and sheds light on how the formation pathways of QDs profoundly influence their functional properties.

## 4 Experimental Section

### Materials

Sulfur (S, 99.998%), oleylamine (OAm, 99%), trioctylphosphine (TOP, 97%), dichloromethane (99.8%), titanium diisopropoxide bis(acetylacetonate) (75 wt.%), tetraethyl orthosilicate (98%), zinc nitrate hexahydrate (Zn(NO_3_)_2_·6H_2_O, 98%), zinc acetate (98%), and sodium sulfide (Na_2_S, 98%) were purchased from Sigma–Aldrich. Ethyl alcohol (99.9%, anhydrous), acetonitrile (99.5%), and sodium sulfite (Na_2_SO_3_, 97.0%, anhydrous) were purchased from Samchun Chemicals. Indium(III) acetate (In(Ac)_3_, 99.99%), indium(III) iodide (InI_3_, 99.998%), and copper(I) iodide (CuI, 99.998%) were purchased from Alfa Aesar. Ammonium hydroxide (NH_4_OH, 25%, solution), methyl alcohol (99.5%), and *n*‐butanol (99.5%) were purchased from Daejung Chemicals & Metals.

### Synthesis of CuInS_2_ QDs

CIS QDs were synthesized through the Lewis acid–base reactions between metal–OAm complexes and S–OAm complexes. The synthesis method was adapted and further refined based on previously reported protocols.^[^ ^35^, ^53^ ^]^ Metal–OAm complexes were synthesized by heating CuI or InX_3_ (X = I^−^ or Acetate^−^) in OAm at 120 °C for 1 h under vacuum conditions. The final cation precursor solution was prepared by dissolving 0.5 mmol of CuI–OAm and 0.5 mmol of InX_3_–OAm complexes in 4.0 mL of OAm. The S precursor was prepared by dissolving 4.0 mmol of S in 4.0 mL of OAm to form the S–OAm complexes. Subsequently, 1.0 mL of the S–OAm solution was injected into the cation precursor solution containing metal–OAm complexes and heated to 180 °C with a constant heating rate (15 °C min^−1^). Then, the reaction mixture was heated at 180 °C for 30 min for the growth of QDs. The products were purified by the standard centrifugation method (4000 rpm, 5 min, 3 cycles) with chloroform (2.0 mL) as the good solvent and ethyl alcohol (38.0 mL) as the poor solvent. During the first centrifugation cycle, 0.25 mL of TOP was additionally added to remove any unreacted sulfur precursor. The final products were obtained by dispersing the precipitate in non‐polar solvents such as hexane or dichloromethane.

### Simultaneous In Situ SAXS and WAXS Analyses

Simultaneous in situ SAXS and WAXS analyses were performed at the 9A beamline of the Pohang Accelerator Laboratory (PAL) in Korea, utilizing MX170‐HS (for SAXS) and MX170‐LS (for WAXS) detectors (Rayonix). The X‐ray energy was set at 19.81 keV. The sample‐to‐detector distances for SAXS and WAXS were 2.0 and 0.24 m, respectively, resulting in the wave vector (*q*) range of 0.01–0.57 Å^−1^ for SAXS and the 2 theta range of 20–65° for WAXS. Each measurement was taken at 30 s intervals, and the beam exposure time and integration time were 15.0 and 5.0 s, respectively. 1D scattering profiles were obtained by azimuthally averaging the 2D scattering images. For the measurements, the reaction mixture was prepared for QD synthesis following the same protocol used in CIS QD synthesis. A small portion of the reaction mixture was quickly extracted, immediately sealed in a capillary tube using paraffin film, and then placed on a heating mount. As soon as the measurement started, the temperature was raised from room temperature to 180 °C with the constant heating rate. In this work, the first recorded in situ SAXS and WAXS patterns at room temperature are denoted as 0 min. SAXS and WAXS patterns were recorded simultaneously. A scattering pattern of the pure OAm solvent was acquired and utilized as the background reference. All SAXS results were analyzed after background subtraction. The background subtraction was performed using the equation presented below:

(1) $$I\left(q\right)=\frac{C\left(q\right)-C_{d}\left(q\right)}{T\left(\lambda \right)t}-\frac{C_{b}\left(q\right)-C_{d}\left(q\right)}{T_{b}\left(\lambda \right)t_{b}}$$

where *C*(*q*), *C_b_* (*q*), *C_d_* (*q*), *T*(*λ*), *t*, *T_b_* (*λ*), and *t_b_* represent the detected scattering intensity, the scattering of background, a dark current, the absorption, the exposure time and the background absorption, and background exposure time, respectively.

Radii of gyration (*R*_g_) were estimated using the Guinier approximation with SASfit software. The values were calculated from a linear fit of ln[*I*(*q*)] versus *q* ^2^ within sufficiently small scattering ranges (*qR*_g_< 1.3). This method is a standardized approach for estimating *R*_g_, as established in early literature.^[^ ^85^ ^]^

### Extended X‐Ray Absorption Fine Structure (EXAFS) Analysis

EXAFS data were collected at the PLS‐II 10C beamline of the Pohang Accelerator Laboratory (PAL) in South Korea. Powdered samples were evenly spread and sealed within polyimide substrates. Cu *K*‐edge and In *K*‐edge EXAFS spectra were acquired at room‐temperature. Energy calibration was carried out by simultaneously capturing spectra from reference samples (metal foils). Data processing and fitting were conducted using Athena 0.9.26 and Artemis 0.9.26 software. For the fitting, variables such as the amplitude reduction factor (*S_0_* ^2^), bond lengths (*R*, Å) were allowed to vary. Considering the crystal structure, the tetrahedral geometry was considered for both Cu─S and In─S.

### Statistical Analysis

The size distribution of CIS QDs from TEM images was analyzed using ImageJ 1.54f software. No pre‐processing of the data was applied. The sample size (*n*) was 200. Assuming the CIS QD particles are spherical, their areas were approximated, and diameters were calculated based on the area data.

## Conflict of Interest

The authors declare no conflict of interest.

## Supporting information



Supporting Information

## Data Availability

The data that support the findings of this study are available in the supplementary material of this article.
