# Supplementary material for: Unveiling Formation Pathways of Ternary I–III–VI CuInS2 Quantum Dots and Their Effect on Photoelectrochemical Hydrogen Generation
Source: Adv Sci (Weinh). 2025 May 28;12(31):e00829. doi: 10.1002/advs.202500829 (PMC12376712; doi:10.1002/advs.202500829)
Supplement: Supplementary file 1 — Supporting Information [file ADVS-12-e00829-s001.pdf]

ADVANCED SCIENCE
Open Access

Supporting Information

for *Adv. Sci.*, DOI 10.1002/advs.202500829

Unveiling Formation Pathways of Ternary I–III–VI $CuInS_2$ Quantum Dots and Their Effect on Photoelectrochemical Hydrogen Generation

Hyo Cheol Lee, Hwapyong Kim, Kiwook Kim, Kyunghoon Lee, Wookjin Chung, Seung Beom Ha, Minseo Kim, Eonhyoung Ahn, Shi Li, Seunghyun Ji, Gyudong Lee, Hyeonjong Ma, Sung Jun Lim, Hongsoo Choi, Jae-Yup Kim, Hyungju Ahn, Su-Il In* and Jiwoong Yang**

Supporting Information

Unveiling Formation Pathways of Ternary I–III–VI $CuInS_2$ Quantum Dots and Their Effect on Photoelectrochemical Hydrogen Generation

Hyo Cheol Lee,† Hwapyong Kim,† Kiwook Kim,† Kyunghoon Lee, Wookjin Chung, Seung Beom Ha, Minseo Kim, Eonhyoung Ahn, Shi Li, Seunghyun Ji, Gyudong Lee, Hyeonjong Ma, Sung Jun Lim, Hongsoo Choi, Jae-Yup Kim, Hyungju Ahn, Su-Il In,* and Jiwoong Yang**

H. C. Lee, H. Kim, K. Kim, K. Lee, W. Chung, M. Kim, E. Ahn, S. Li, S. Ji, H. Ma, Prof. S.-I. In, Prof. J. Yang
Department of Energy Science and Engineering, Daegu Gyeongbuk Institute of Science and Technology (DGIST), Daegu 42988, Republic of Korea
*E-mail: insuil@dgist.ac.kr (S.-I. In), jiwoongyang@dgist.ac.kr (J. Yang)

H. C. Lee
Department of Chemistry, Hong Kong University of Science and Technology (HKUST), Kowloon 999077, Hong Kong SAR, Hong Kong

S. B. Ha
Department of Chemical Engineering, Dankook University, Yongin 16890, Republic of Korea

Dr. G. Lee, Dr. S. J. Lim
Division of Nanotechnology, Daegu Gyeongbuk Institute of Science and Technology (DGIST), Daegu 42988, Republic of Korea

Dr. G. Lee, Prof. H. Choi
DGIST-ETH Microrobotics Research Center, Daegu Gyeongbuk Institute of Science and Technology (DGIST), Daegu 42988, Republic of Korea

Prof. H. Choi
Department of Robotics and Mechatronics Engineering, Daegu Gyeongbuk Institute of science and Technology (DGIST), Daegu 42988, Republic of Korea

Prof. J.-Y. Kim
Department of Chemical Engineering, Konkuk University, Seoul 05029, Republic of Korea

Dr. H. Ahn
Pohang Accelerator Laboratory, Pohang University of Science and Technology (POSTECH), Pohang 37673, Republic of Korea
*E-mail: hyungju@postech.ac.kr (H.A.)

Prof. S.-I. In, Prof. J. Yang
Energy Science and Engineering Research Center, Daegu Gyeongbuk Institute of Science and Technology (DGIST), Daegu 42988, Republic of Korea

† H. C. Lee, H. Kim, and K. Kim contributed equally to this work.

1. Supporting Methods

Material Characterization. Absorption spectra were measured using a Cary 5000 UV–Vis–NIR spectrophotometer (Agilent Technologies). X-ray diffraction (XRD) patterns were recorded with a MiniFlex 600 benchtop diffractometer (Rigaku), employing Cu-Kα radiation as the X-ray source. Transmission electron microscope (TEM) and scanning electron microscope (SEM) images were taken with a Tecnai G2 F20 TWIN TMP (FEI) and a S-4700 field emission scanning electron microscope (Hitachi), respectively. Raman spectra were measured using an inVia Qontor (Renishaw), with the laser wavelength set at 532 nm. Inductively coupled plasma optical emission spectroscopy (ICP-OES) measurements were carried out with an iCAP7400DUO (Thermo Scientific). Photoluminescence (PL) spectra were obtained using an FS5 Spectrofluorometer (Edinburgh Instruments). Time-resolved photoluminescence (TRPL) measurements were conducted using an FS5 Spectrofluorometer (Edinburgh Instruments). Data were collected at the PL emission center of each sample. Fourier Transform Infrared (FTIR) spectra were obtained using a Cary 660 FTIR spectrometer (Agilent). X-ray photoelectron spectroscopy (XPS) was performed using a ESCALAB 250Xi system (Thermo Scientific) with Al Kα radiation (1486.6 eV). The C 1s peak (binding energy: 284.8 eV) served as a reference to eliminate the charge effect.

Mott–Schottky curves for $CuInS_2$ (CIS) quantum dots (QDs) were recorded with an Autolab PGSTAT302N potentiostat (Metrohm), employing a non-aqueous three-electrode system. The working electrode was fabricated with CIS QDs deposited on indium tin oxide (ITO) glass. A platinum (Pt) wire functioned as the counter electrode, and an Ag/Ag^+ electrode, immersed in an acetonitrile solution containing 0.1 M tetra-*n*-butylammonium perchlorate (TBAP) and 0.01 M silver nitrate ($AgNO_3$), served as the reference electrode. The chosen electrolyte was a 0.1 M TBAP solution in acetonitrile. To reduce the effects of oxygen, this electrolyte was pre-treated with argon gas for 30 min before each experiment. Mott–Schottky measurements were carried out across a frequency range of 100 kHz to 0.1 Hz, with a signal amplitude set at 5 mV.

From the slopes of these Mott–Schottky curves, the carrier concentration (N) was calculated, using an equation detailed below.

$$N\ (\text{cm}^{-3}) = \left(\frac{2}{e\varepsilon\varepsilon_0}\right)\left[\frac{d\left(\frac{1}{C^2}\right)}{dV}\right]^{-1}$$

where e, ε, ε_0, C, and V denote the elementary charge (1.6×10^{-19} C), the relative dielectric constant of the semiconductor (10.2 for bulk CIS),[S1] the vacuum permittivity (8.8×10^{-12} F m^{-1}), the interfacial capacitance (F cm^{-2}), and the applied voltage (V), respectively.

The SCLC curves for both electron-only and hole-only devices were analyzed in dark conditions using a Keithley 2636B source meter. We calculated the trap density (n_{trap}) of these devices using the equation provided below.

$$n_{\text{trap}}\ (\text{cm}^{-3}) = \frac{2\varepsilon\varepsilon_0 V_{\text{TFL}}}{eL^2}$$

where V_{TFL} (V) is the trap-field limit voltage and L is the film thickness of the CIS QDs measured by Park XE7 atomic force microscopy (Park Systems).

The carrier mobility (μ) of the devices was determined using the following equation.

$$\mu\ (\text{cm}^2\ \text{V}^{-1}\ \text{s}^{-1}) = \frac{8J_{\text{D}}L^3}{9\varepsilon\varepsilon_0 V^2}$$

where J_D (mA cm^{-2}) is the dark current density.

Electron- and hole-only devices were prepared as follows. Initially, ITO glass underwent ultrasonic cleaning in detergent, acetone, and ethanol, each for 15 min. After drying the glass with argon flow, it was subjected to UV/O_3 treatment (UV/ozone cleaner, Omniscience, Korea) for 10 min. For the electron-only devices, a CIS QD film was spin-coated onto the ITO glass at 2,000 rpm for 15 s and then annealed at 120 °C for 10 min. Subsequently, a ZnO film was spin-coated onto the CIS QD film at the same speed and duration, followed by annealing at 120 °C for 10 min. A silver (Ag) electrode was then thermally evaporated onto the ZnO film at a rate of 1 Å s^{-1}. In contrast, for the hole-only devices, a poly(3,4-ethylenedioxythiophene)-poly(styrenesulfonate) (PEDOT:PSS) film was spin-coated onto the ITO glass at 3,000 rpm for

30 s, followed by annealing at 180 °C for 20 min. The CIS film and Ag electrode for these devices were prepared using the same parameters as for the electron-only devices.

Preparation of CIS QD-Sensitized TiO_2 Photoanodes. CIS QD-sensitized TiO_2 photoanodes were fabricated using the previously reported method.[S2] A 3×4 cm piece (for 4 electrode samples) of fluorine-doped tin oxide glass (FTO, TEC-A7; Pilkington, United Kingdom) was cleaned by sonication in acetone and then in ethanol for 15 min each. The washed FTO glass was treated with a UV/O_3 cleaner for 15 min. Next, an electron transfer layer (ETL) was deposited on the FTO glass by spin-coating a 7.5 wt% solution of titanium diisopropoxide bis(acetylacetonate) in *n*-butanol inside an Ar-filled glovebox. This ETL ensured charge separation by suppressing the recombination of photogenerated holes in the CIS QDs. The spin-coated FTO glass was then annealed in air at 450 °C for 10 min. For the TiO_2 transparent layer coating, a commercial TiO_2 paste (Ti-Nanoxide T/SP; Solaronix, Switzerland) was applied to the FTO glass using the doctor blade technique and then annealed in air at 500 °C for 30 min. Similarly, a TiO_2 scattering layer was applied over the TiO_2 transparent layer-coated FTO glass. The same commercial TiO_2 paste was spread onto the FTO glass using the doctor blade technique, followed by annealing at 500 °C for 30 min in air.

To sensitize CIS QDs onto a TiO_2 photoelectrode, a dense TiO_2 film-coated FTO glass was immersed in a dichloromethane solution dispersed with CIS–InI_3 or CIS–$In(Ac)_3$ QDs for 24 h. The TiO_2/QDs film-coated FTO glass was washed with methanol and dried under a N_2 gas flow. For surface passivation of the TiO_2/QD film, a zinc sulfide (ZnS) layer was coated using the conventional successive ionic layer adsorption and reaction (SILAR) method for three cycles. The TiO_2/QDs film was soaked in a 0.1 M zinc acetate aqueous solution for 1 min, followed by washing with methanol. Then, the cation precursor-treated TiO_2/QDs film was soaked in a 0.1 M sodium sulfide methanolic solution for 1 min, followed by washing with methanol. This SILAR process was repeated three times in total. To improve the stability of the CIS QD-

sensitized TiO_2 photoanodes, an additional SiO_2 passivation layer was applied over the ZnS-passivated TiO_2/QDs film using the chemical bath deposition (CBD) method. The TiO_2/CIS QDs/ZnS film was immersed in a solution of 0.01 M tetraethyl orthosilicate and 0.1 M NH_4OH in ethanol for 70 min. Next, the TiO_2/CIS QDs/ZnS/SiO_2 film was cleaned by rinsing with distilled water and dried under vacuum at 40 °C for 30 min.

Preparation of CIS QD-Sensitized $BiVO_4$ Photoanodes. FTO glasses were cleaned by sonication in acetone and then in ethanol for 15 min each and treated with UV/O_3 for 20 min. To electrodeposit the BiOI film onto the FTO glasses, a 0.04 M $Bi(NO_3)_3$ (Sigma-Aldrich) and 0.4 M KI (Sigma-Aldrich) aqueous solution was prepared and adjusted to pH 1.7 using HNO_3. Then, 0.23 M *p*-benzoquinone (Sigma-Aldrich) was dissolved in 20 mL of ethanol and added to the BiOI solution. The electrodeposition was performed by applying +0.1 V in a three-electrode system at room temperature for 4 min (working electrode: Pt holder, reference electrode: Ag/AgCl, counter electrode: Pt mesh). The electrode was then rinsed with deionized water to remove any unreacted substances. Then, a 0.2 M vanadyl acetylacetonate (Sigma-Aldrich) solution in dimethyl sulfoxide (Sigma-Aldrich) was dropped onto the BiOI (vanadyl acetylacetonate 100 μL per 1 cm^2 BiOI). The BiOI was then heated at 450 °C for 2 h. After annealing, the $BiVO_4$ photoelectrode was soaked in 1 M NaOH to remove excess V_2O_5 from the $BiVO_4$ surface. The sensitization of CIS QDs and ZnS/SiO_2 passivation followed the same processes as the fabrication process of TiO_2/CIS QDs/ZnS/SiO_2 photoanodes.

Photoelectrochemical (PEC) Characterization of CIS QD-Sensitized TiO_2 Photoanodes. The PEC performance and properties were analyzed using a VSP potentiostat (BioLogic) and a 450 W Xenon solar simulator (94043A, Newport) equipped with an AM 1.5G filter (81388, Newport). The active area of the photoanode (0.20 ± 0.02 cm^2) was measured with a CCD camera. All PEC tests were conducted using a three-electrode system with a mercury/mercury

oxide (Hg/HgO) electrode, Pt mesh, and CIS QD-sensitized TiO_2 photoanodes serving as the reference electrode, counter electrode, and working electrode, respectively. The electrolyte consisted of a 0.25 M sodium sulfide (Na_2S) and 0.35 M sodium sulfite (Na_2SO_3) aqueous solution (pH ~12.5). The hydrogen evolution reaction was tested under 1.0 sun (100 mW cm^{-2}) conditions using a 450 W Xenon lamp equipped with an AM 1.5G filter, and an applied potential of 0.6 V_{RHE} to the photoanodes using the chronoamperometry method with the potentiostat. The amount of hydrogen produced was analyzed by gas chromatography (GC, 7890B; Agilent, USA) equipped with a MoleSieve 5A packed column (6 ft in length, 2.1 mm inner diameter).

Photocurrent density−voltage (J−V) plots were measured from −0.3 to +1.2 V_{RHE} with 20 mV s^{-1} scan rate and 1.0 sun (100 mW cm^{-2}) condition. The applied bias photon-to-current efficiency (ABPE) was converted from photocurrent density and applied potential of J−V plots by the following Equation.[S3]

$$\text{ABPE (\%)} = \left(\frac{J_{\text{ph}} \times (V_{\text{rev}} - V_{\text{app}})}{P_{\text{in}}}\right) \times 100\%$$

where J_{ph} (mA cm^{-2}) is the measured photocurrent density, V_{rev} is the standard-state reversible potential of water (1.23 V), V_{app} (V) is the applied external potential vs reversible hydrogen electrode (RHE), and P_{in} is the power density of the incident light (100 mW cm^{-2}) of the 450 W solar simulator.

The incident photon-to-current efficiency (IPCE) analysis was conducted at 0.6 V_{RHE} applied potential and 1.0 sun by the 450 W solar simulator with AM 1.5G filter and monochromator (Cornerstone™ 130 1/8m; Newport). IPCE (%) was calculated by the Equation.[S3]

$$\text{IPCE (\%)} = \frac{J_{\text{ph}} \times h \times c}{P_{\text{mono}} \times e \times \lambda} \times 100$$

where J_{ph} (mA cm^{-2}) is the measured from chronoamperometry mode applying 0.6 V_{RHE}, h is Plank's constant (6.626×10^{-34} J s), c is the speed of light (3.0×10^{8} m s^{-1}), P_{mono} (mW cm^{-2}) is the power density of the fixed incident light of monochromatography.

Nyquist plot was measured at 0.6 V_{RHE} and ±10 mV sinus amplitude by the potentiostat. A frequency range of Nyquist plot measurement is from 100 mHz to 100 kHz.

Mott–Schottky plots were measured in range from +1.2 to −0.3 V_{RHE} at the dark condition with a 1 kHz frequency. Donor concentration (N_d) and flat band potential (V_{FB}) of the photoanodes were measured from the slope of the Mott–Schottky plot and extrapolated *x*-axis intercept, respectively by following equation.[S4]

$$\frac{1}{C^2} = \left(\frac{2}{e\varepsilon\varepsilon_0 N_\mathrm{d}}\right)\left[V - V_\mathrm{FB} - \frac{k_\mathrm{B}T}{e}\right]$$

where C (F cm^{-2}) is the capacitance of the space-charge layers, V (V_{RHE}) is the applied potential of Mott–Schottky analysis, N_d (cm^{-3}) is the donor concentration, k_B is Boltzmann's constant (1.38×10^{-23} J K^{-1}), T is the room temperature (298 K), e is the elementary charge (1.602×10^{-19} C), ε_0 is the vacuum permittivity (8.86×10^{-12} F m^{-1}), and ε is the dielectric constant for bulk CIS (10.2).[S1]

Electron lifetime of the photoanodes was analyzed by surface photovoltage (SPV) measurements and the open circuit voltage decay (OCVD) technique. SPV measurements were conducted with a custom-built apparatus that included a monochromator, light shutter, and potentiostat, over a wavelength range of 300 to 900 nm. To measure OCVD, the photoanodes were illuminated by the 1.0 sun incident light during 20 s then light was suddenly blocked with measuring photovoltage decay by open circuit voltage measurement mode of the potentiostat. The electron lifetime is measured by following equation.

$$\tau_\mathrm{e}\ (\mathrm{s}) = \left(\frac{k_\mathrm{B}T}{e}\right)\left(\frac{\mathrm{d}V_\mathrm{OC}}{\mathrm{d}t}\right)^{-1}$$

where V_{OC} (V) is the open circuit voltage.

The amount of hydrogen was measured under 1.0 sun illumination condition with applying 0.6 V_{RHE} by the potentiostat, the 450 W solar simulator, and gas chromatography (7890B; Agilent) equipped with molecular sieve 5A column (80474-810, 6 inch, 1/8 inch, SS packed type, Restek).

Faradaic efficiencies (*FE*) and hydrogen yields were calculated using following equations.

$$FE\ (\%) = \frac{Q_{\mathrm{exp}}}{Q_{\mathrm{th}}} \times 100$$

$$\text{Hydrogen yields (mol)} = \frac{Q_{\mathrm{th}} \times FE}{Z \times F} \times 100$$

where Q_{exp} (C) is the experimental charge converted to hydrogen fuel, Q_{th} (C) is the theoretical charge passed during the photoelectrochemical experiment, Z is the number of electrons for hydrogen (1.20×10^{24} mol^{-1}) and F is the faradaic constant (96,485 C mol^{-1}).

2. Supporting Figures

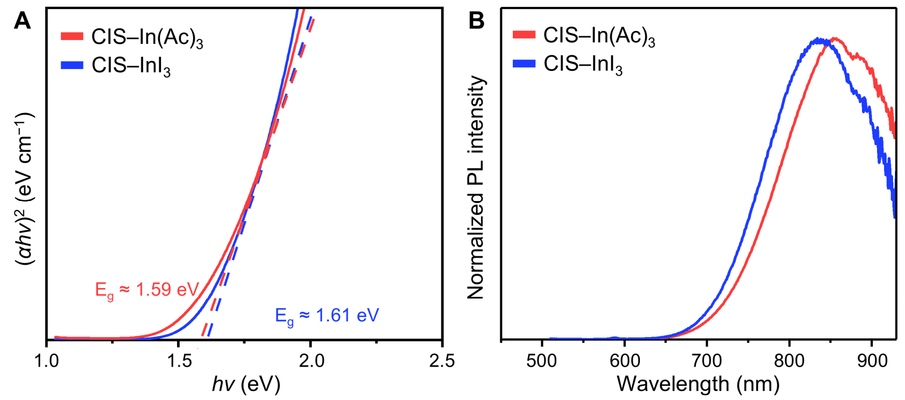

Figure S1. A) Tauc plots of two types of CIS QDs, obtained from Figure 1B. B) PL spectra of CIS QDs. The peak emission center of CIS−In(Ac)$_3$ QDs is ~855 nm (1.45 eV), while that of CIS−InI$_3$ QDs is ~840 nm (1.47 eV).

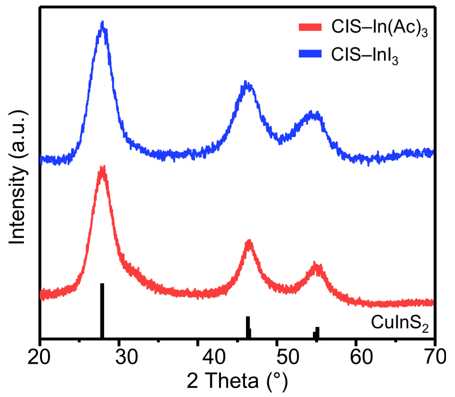

Figure S2. XRD patterns of CIS−In(Ac)$_3$ and CIS−InI$_3$ QDs. Reflections of bulk $CuInS_2$ (JCPDS No. 47-1372) are presented as black bars. The crystallite sizes, estimated from the Scherrer equation, are ~5.8 nm for both CIS−In(Ac)$_3$ and CIS−InI$_3$ QDs.

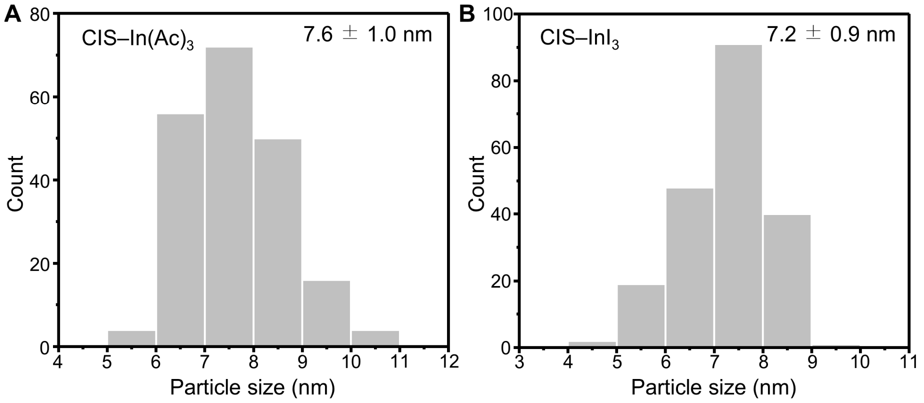

Figure S3. Histograms showing the size distribution of A) CIS−In(Ac)$_3$ and B) CIS−InI$_3$ QDs (*n* = 200).

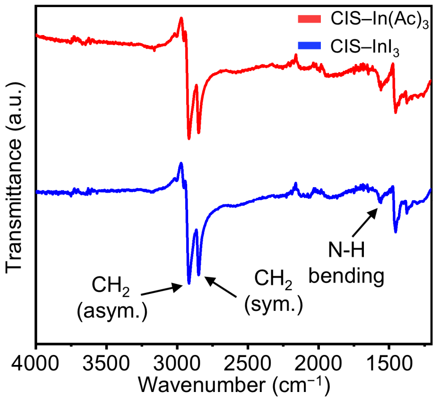

Figure S4. FTIR spectra of CIS−In(Ac)$_3$ and CIS−InI$_3$ QDs.

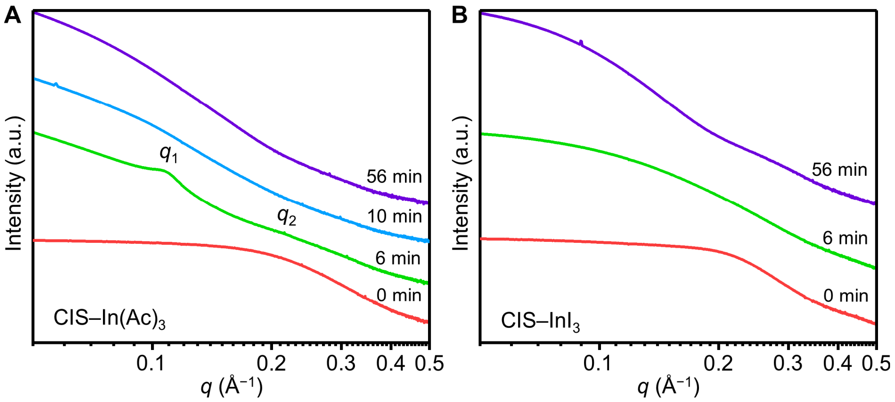

Figure S5. The selected in situ small angle X-ray scattering (SAXS) patterns acquired from A) Figure 2A and B) Figure 2B, showing several representative reaction stages. The scattering intensities are plotted using a logarithmic scale.

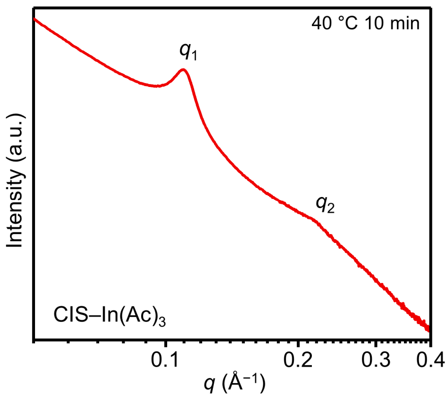

Figure S6. SAXS pattern of the reaction mixture for the synthesis of CIS−In(Ac)$_3$ QDs. The scattering intensity is plotted using a logarithmic scale.

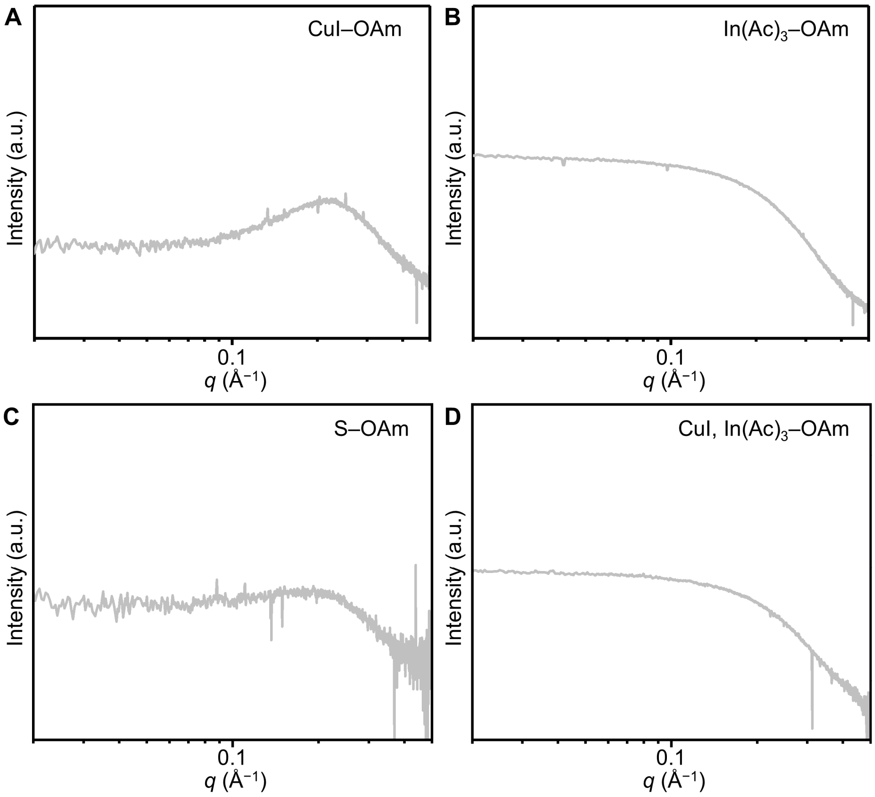

Figure S7. Ex situ SAXS patterns of the OAm solution containing A) CuI−OAm, B) $In(Ac)_3$−OAm, C) S−OAm, and D) CuI, $In(Ac)_3$−OAm complexes. The scattering intensities are plotted using a logarithmic scale.

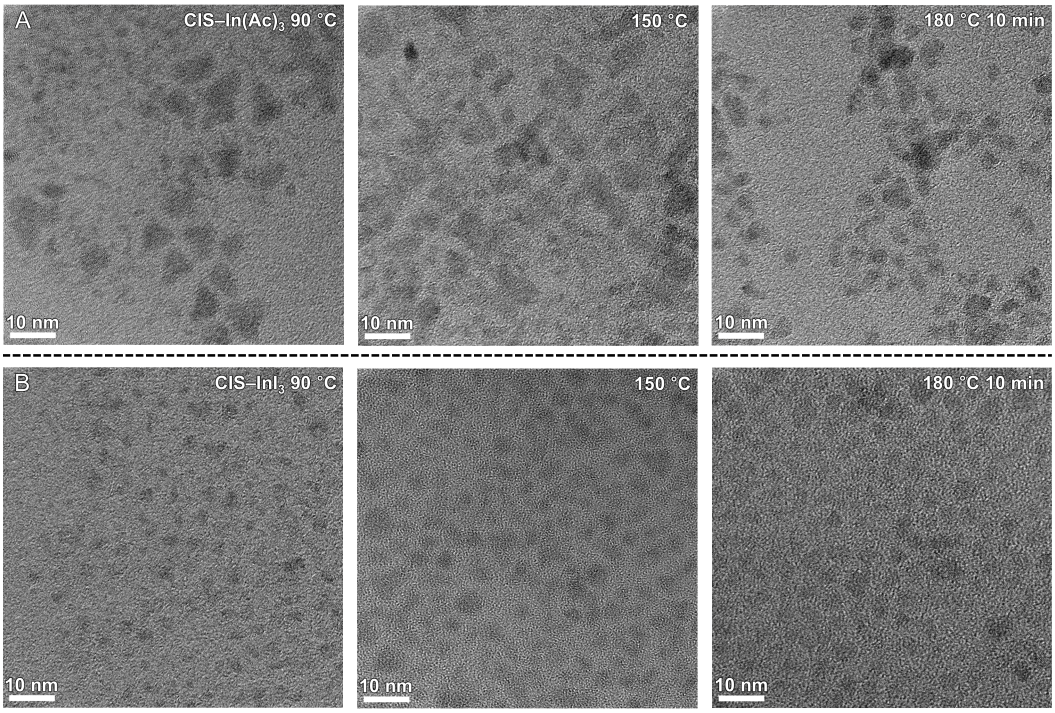

Figure S8. TEM images of the intermediates during the synthesis of A) CIS−$In(Ac)_3$ and B) CIS−InI_3 QDs.

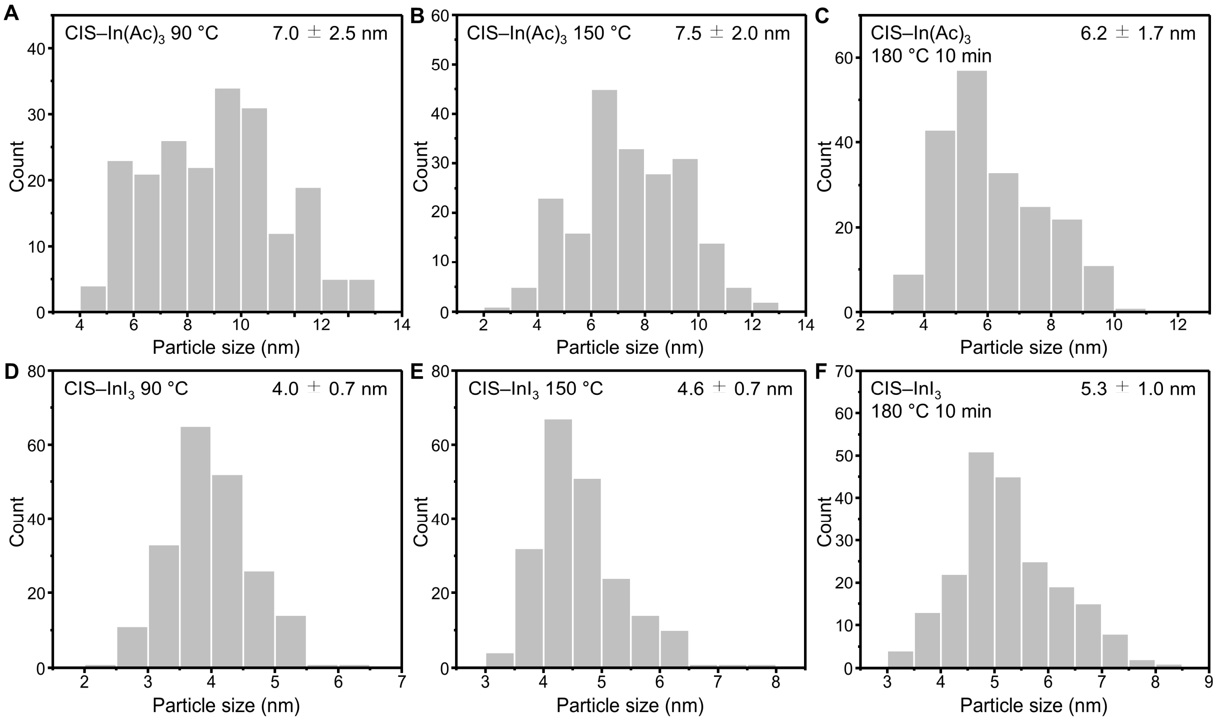

Figure S9. Histograms showing the size distribution of intermediates during the syntheses of A−C) CIS−In(Ac)$_3$ and D−F) CIS−InI$_3$ QDs (*n* = 200).

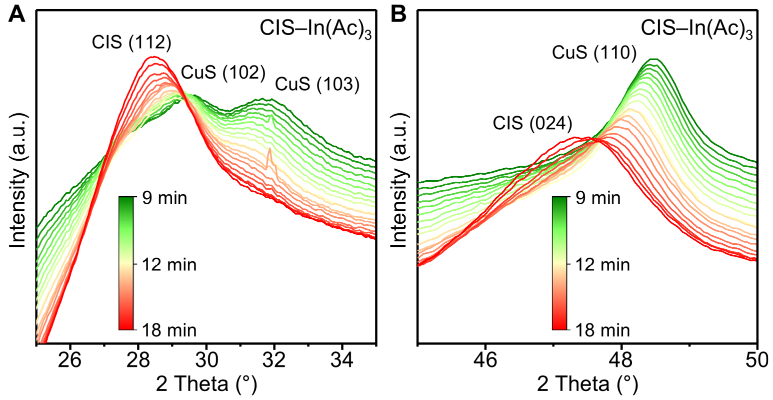

Figure S10. Magnified in situ small angle X-ray scattering (WAXS) patterns on the regime corresponding to A) CuS (102)/CIS (112), CuS (103) and B) CuS (110)/CIS (024) planes during the early stage of CIS−In(Ac)$_3$ QD synthesis (9−18 min).

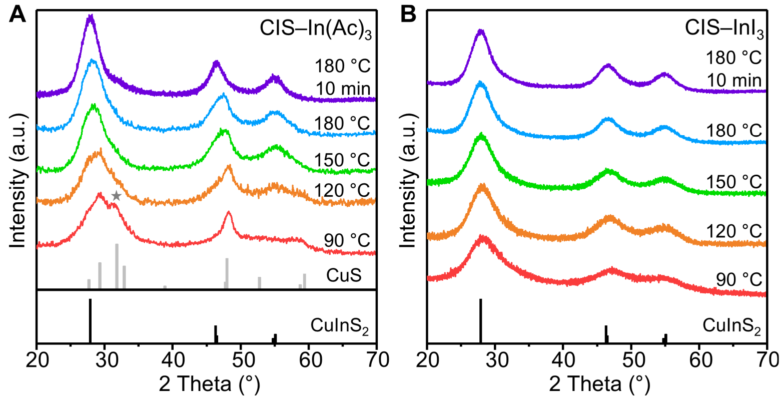

Figure S11. XRD patterns of the intermediates during the synthesis of A) CIS−In(Ac)$_3$ and B) CIS−InI$_3$ QDs. Reflections of Bulk CuS (JCPDS No. 79-2321) and $CuInS_2$ (JCPDS No. 47-1372) are presented as gray and black bars, respectively. A gray star denotes the (103) plane of bulk CuS.

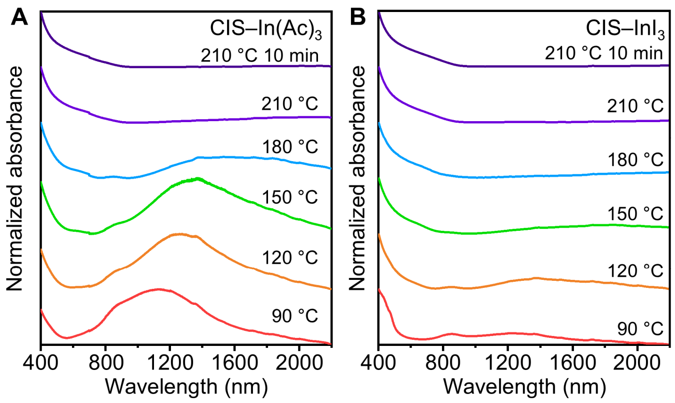

Figure S12. Temporal evolution of absorption spectra during the synthesis of A) CIS–In(Ac)$_3$ and B) CIS–InI$_3$ QDs at an elevated temperature of 210 °C.

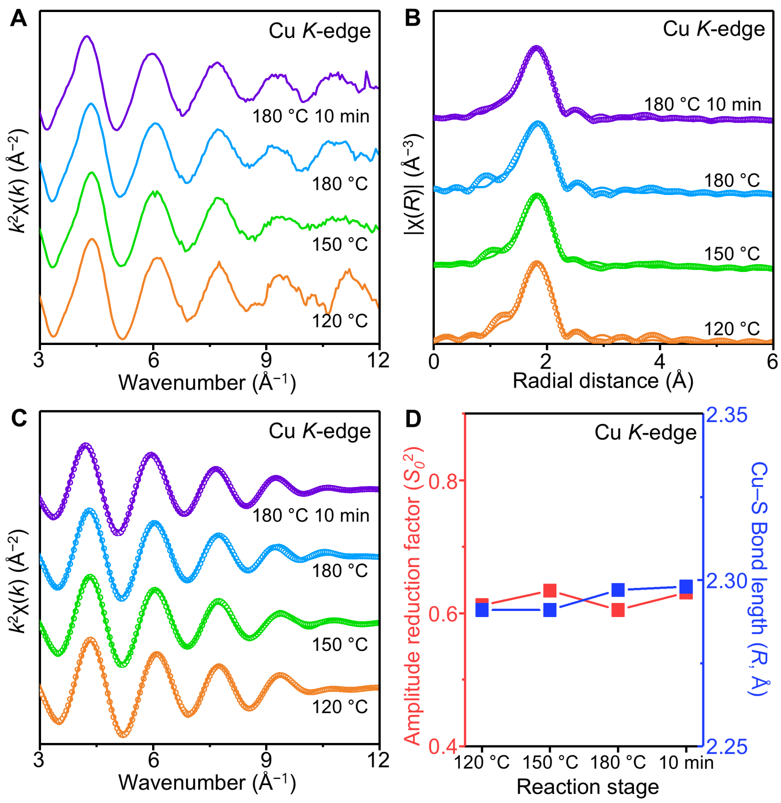

Figure S13. X-ray absorption analysis of the formation of CIS−InI_3 QDs. Cu *K*-edge A) experimental k^2-weighted EXAFS oscillations, B) Fourier-transformed EXAFS spectra, and C) Fourier-filtered EXAFS spectra of intermediates during the synthesis of CIS−InI_3 QDs. D) Cu−S amplitude reduction factor (S_0^2) and bond length (R, Å), estimated by EXAFS fitting analysis. Solid lines represent fitted data while circled patterns represent experimental data.

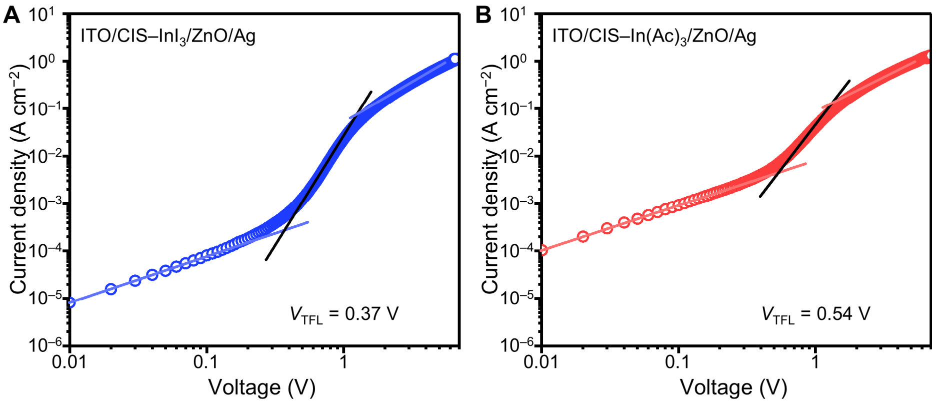

Figure S14. Space charge limited current (SCLC) measurement of the electron-only devices with an architecture of ITO/CIS QDs/ZnO/Ag. Measurement results for A) CIS−InI_3 and B) CIS−$In(Ac)_3$ QD-based devices.

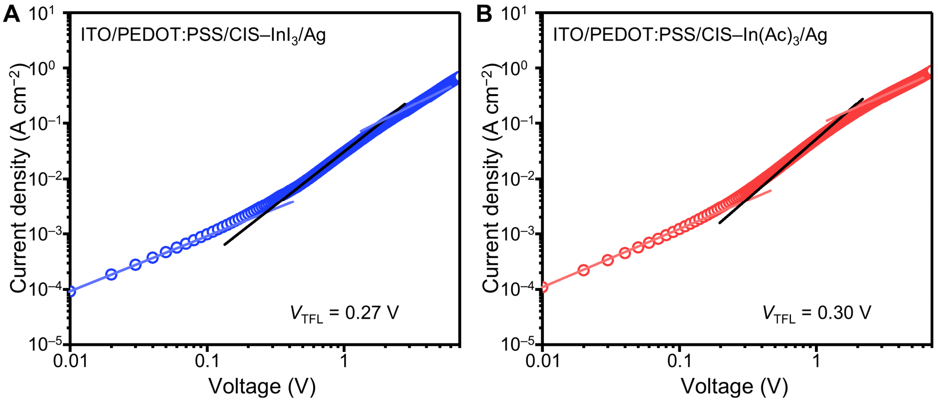

Figure S15. SCLC measurement of the hole-only devices with an architecture of ITO/PEDOT:PSS/CIS QDs/Ag. Measurement results for A) CIS−InI_3 and B) CIS−$In(Ac)_3$ QD-based devices.

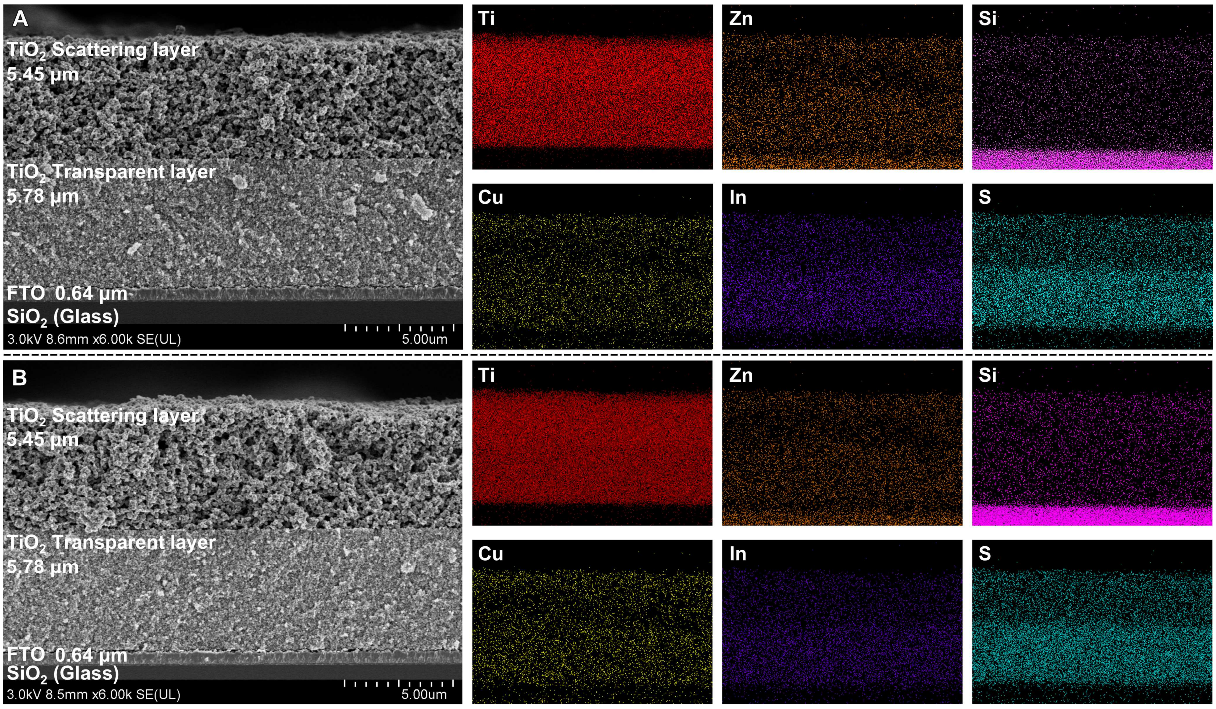

Figure S16. SEM analysis of QD-sensitized photoanodes. Cross-sectional SEM images and corresponding energy dispersive X-ray spectroscopy (EDS) mapping of A) CIS−InI_3 and B) CIS−$In(Ac)_3$ QD-sensitized TiO_2 photoanodes. The thicknesses of each layer for CIS QD-sensitized TiO_2 photoanodes are as follows: TiO_2 scattering layer (5.45 μm), TiO_2 transparent layer (5.78 μm), and FTO (0.64 μm).

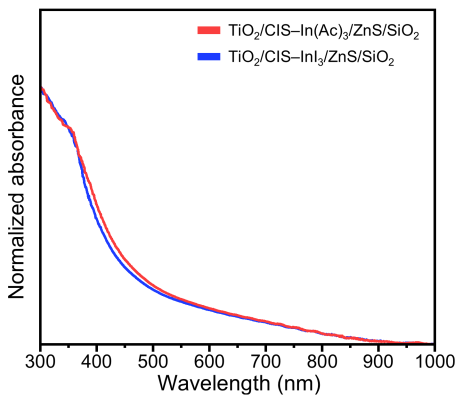

Figure S17. Absorption spectra of TiO_2/CIS–InI_3 QDs/ZnS/SiO_2 and TiO_2/CIS–$In(Ac)_3$ QDs/ZnS/SiO_2 photoanodes.

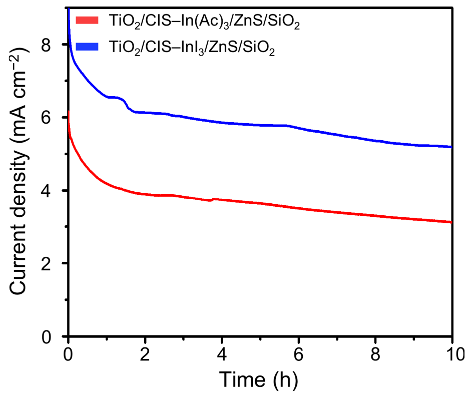

Figure S18. Chronoamperometry test for CIS QD-sensitized TiO_2 photoanodes at 0.6 V_{RHE} for 10 h.

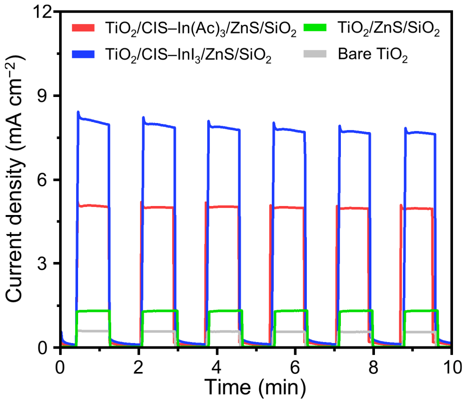

Figure S19. The current density response under chopped-light illumination for CIS QD-sensitized TiO_2 photoanodes at 0.6 V_{RHE}.

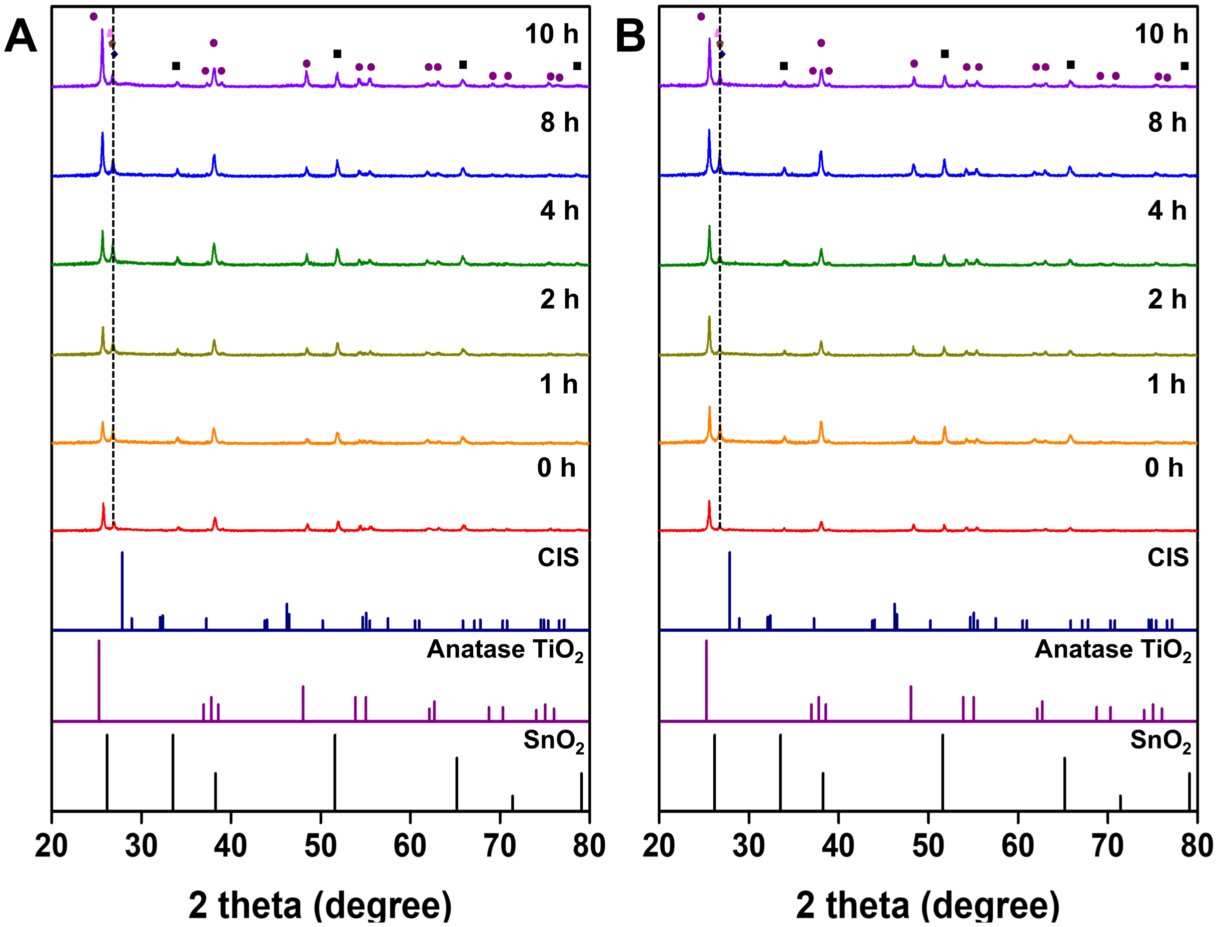

Figure S20. XRD patterns for A) TiO_2/CIS–InI_3 QDs/ZnS/SiO_2 and B) TiO_2/CIS–In(Ac)$_3$ QDs/ZnS/SiO_2 photoanodes as a function of PEC test period. The black dashed lines indicate the position of the (112) plane of the CIS phase. Reflections of bulk $CuInS_2$ (JCPDS No. 47-1372), anatase TiO_2 (JCPDS No. 21-1272), and SnO_2 (JCPDS No. 01-0625) are presented as references.

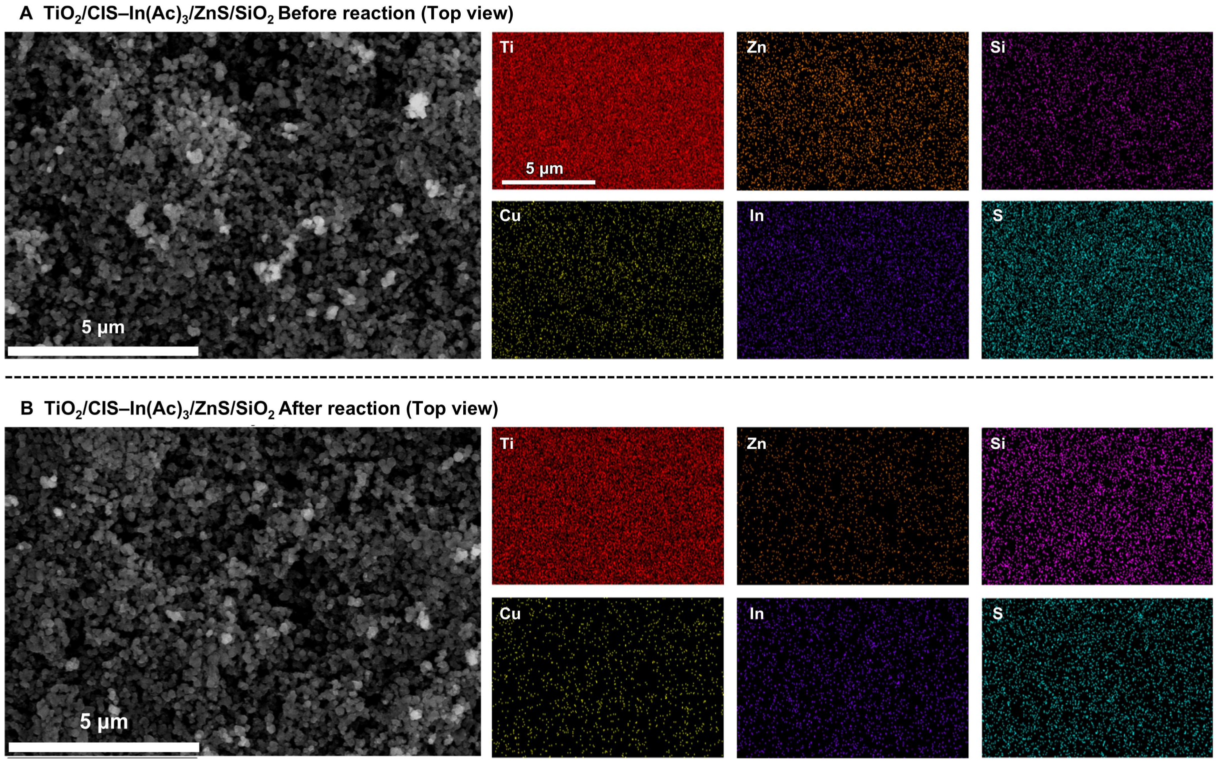

Figure S21. SEM analysis of TiO_2/CIS–In(Ac)$_3$ QDs/ZnS/SiO_2 photoanodes. Top-view SEM images and corresponding EDS mapping of A) before and B) after 10 h of the PEC experiment.

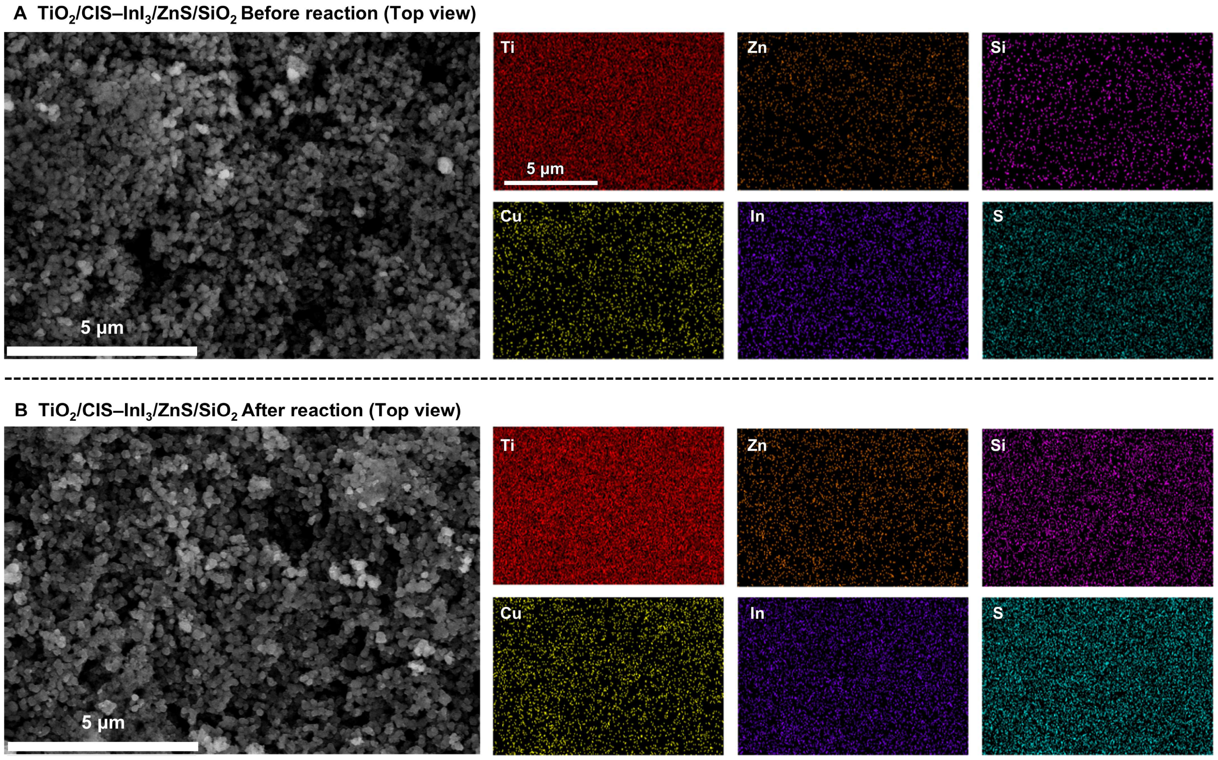

Figure S22. SEM analysis of TiO_2/CIS–InI_3 QDs/ZnS/SiO_2 photoanodes. Top-view SEM images and corresponding EDS mapping of A) before and B) after 10 h of the PEC experiment.

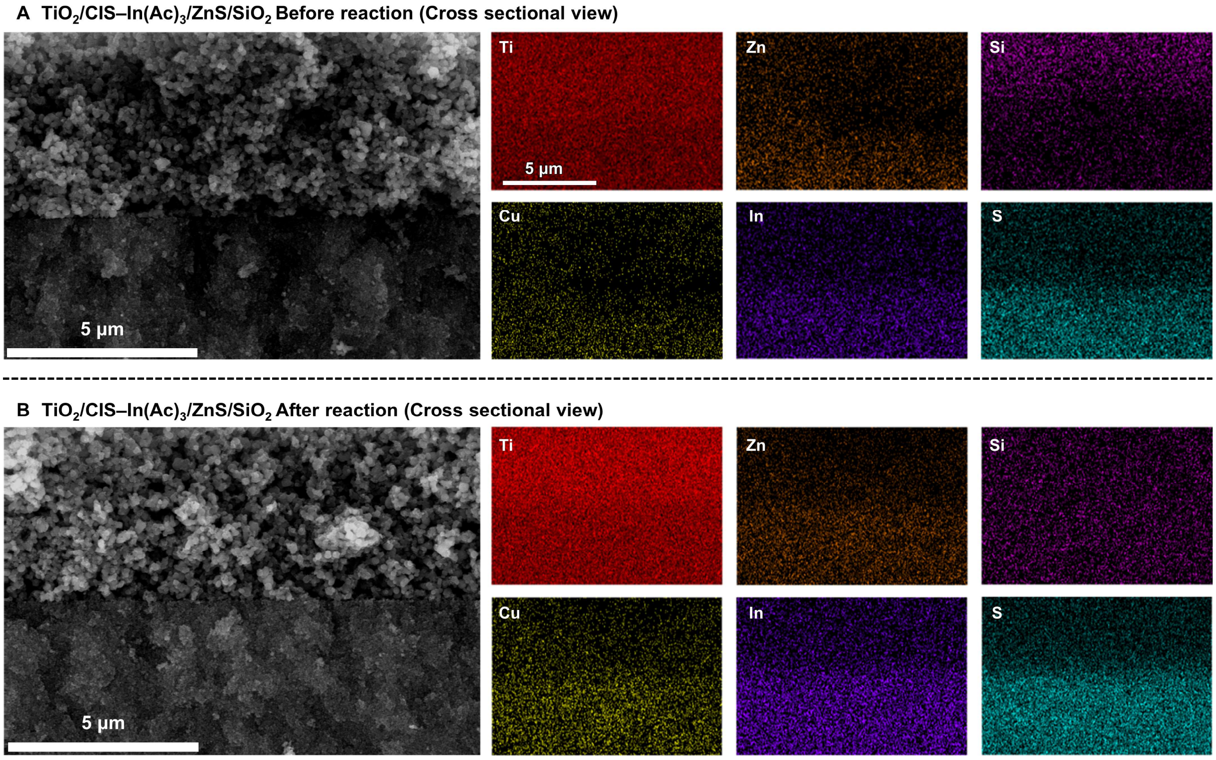

Figure S23. SEM analysis of TiO_2/CIS–In(Ac)$_3$ QDs/ZnS/SiO_2 photoanodes. Cross-sectional SEM images and corresponding EDS mapping of A) before and B) after 10 h of the PEC experiment.

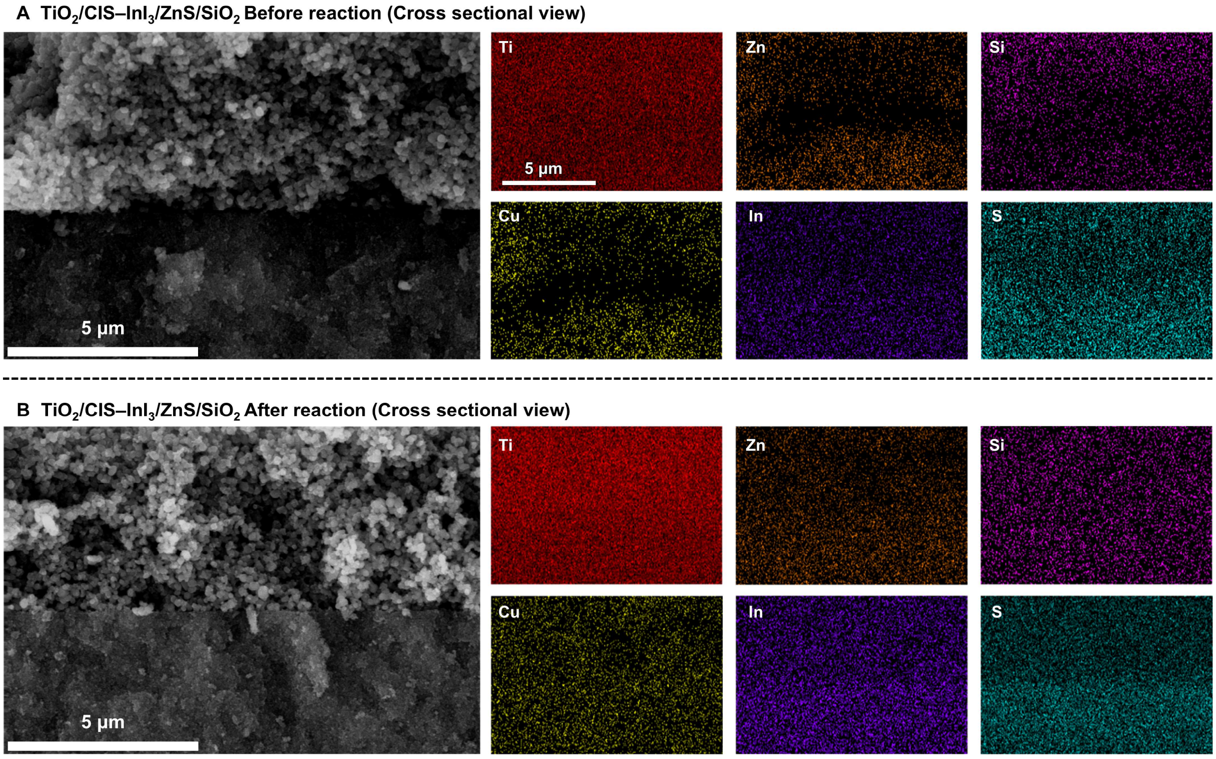

Figure S24. SEM analysis of TiO_2/CIS–InI_3 QDs/ZnS/SiO_2 photoanodes. Cross-sectional SEM images and corresponding EDS mapping of A) before and B) after 10 h of the PEC experiment.

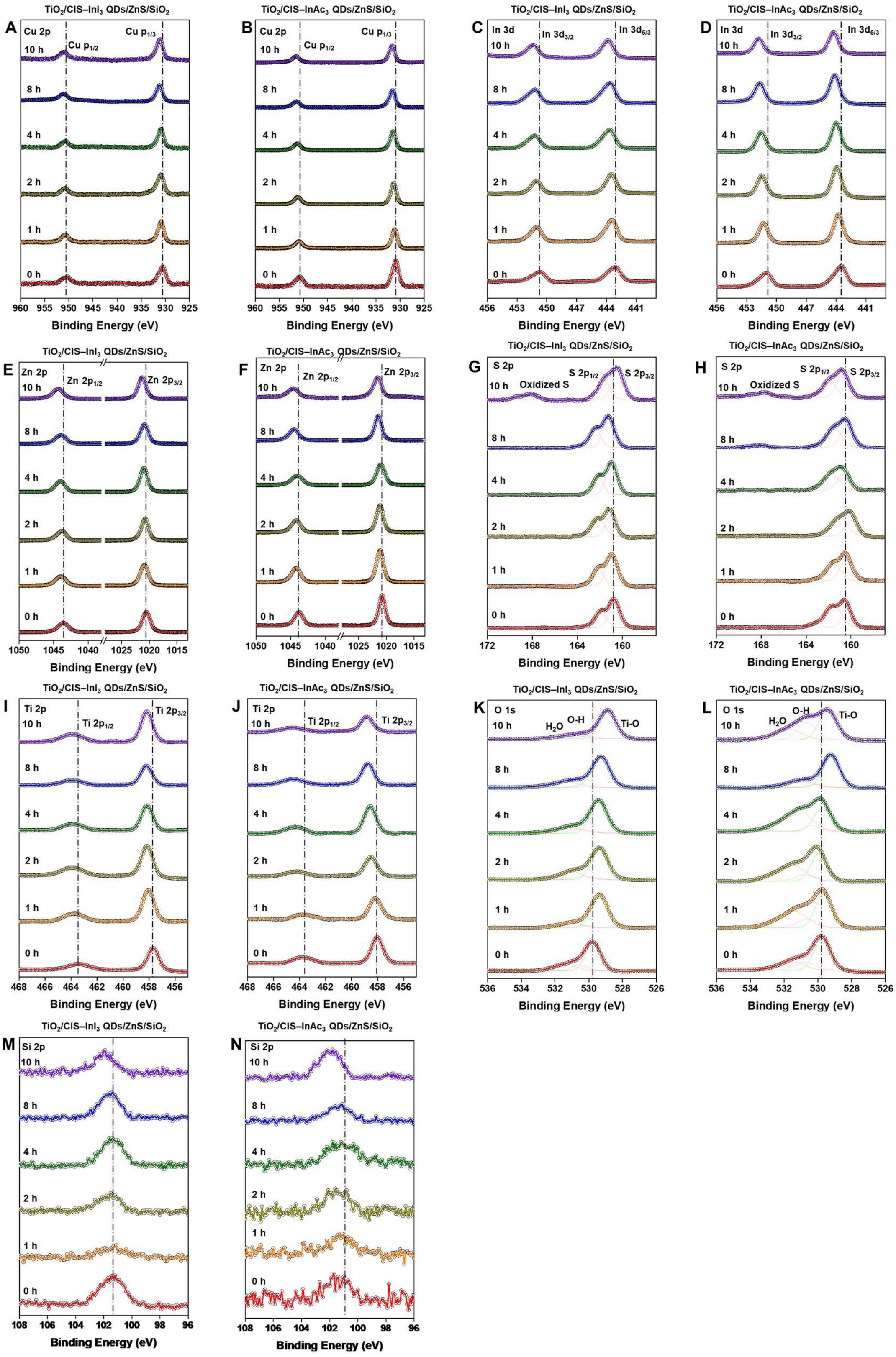

Figure S25. XPS spectra of TiO_2/CIS-InI_3 QDs/ZnS/SiO_2: A) Cu 2p, C) In 3d, E) Zn 2p, G) S 2p, I) Ti 2p, K) O 1s, M) Si 2p and TiO_2/CIS-$InAc_3$ QDs/ZnS/SiO_2: B) Cu 2p, D) In 3d, F) Zn 2p, H) S 2p, J) Ti 2p, L) O 1s, N) Si 2p as a function of PEC test period.

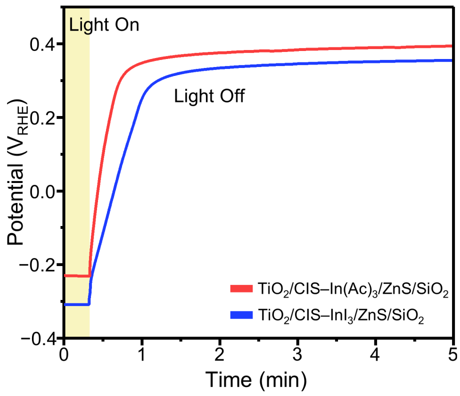

Figure S26. Open circuit voltage decay (OCVD) curves for CIS QD-sensitized TiO_2 photoanodes.

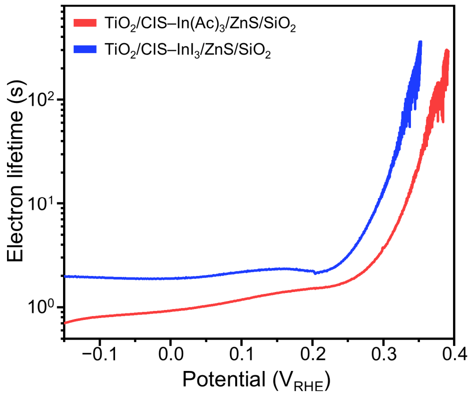

Figure S27. Electron lifetime analysis calculated by the equation of OCVD.

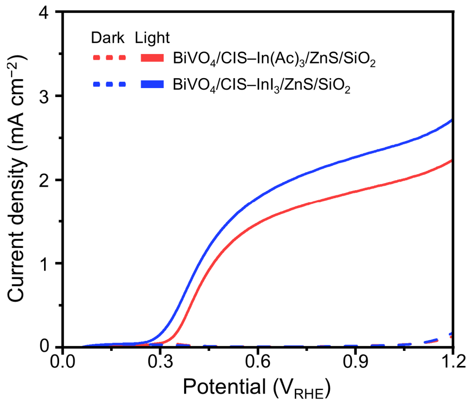

Figure S28. Current–voltage curves of the $BiVO_4$/CIS QDs/ZnS/SiO_2 photoanodes.

3. Supporting Tables

Table S1. Atomic ratio of CIS QDs, estimated by ICP-OES analysis.

Samples	Cu	In
CIS−In(Ac)$_3$ QDs	41.7%	58.3%
CIS−InI$_3$ QDs	39.0%	61.0%

Table S2. Radii of gyration acquired by the Guinier approximation for the synthesis of two CIS QDs as a function of reaction time.

CIS−In(Ac)$_3$ QDs				CIS−InI$_3$ QDs			
**Reaction time (min)**	**Radius of gyration (nm)**	**Reaction time (min)**	**Radius of gyration (nm)**	**Reaction time (min)**	**Radius of gyration (nm)**	**Reaction time (min)**	**Radius of gyration (nm)**
15	4.61	26	4.58	15	1.94	26	2.25
16	4.71	27	4.62	16	2.08	27	2.23
17	4.77	28	4.57	17	2.07	28	2.27
18	4.44	29	4.50	18	2.18	29	2.26
19	4.67	30	4.57	19	2.14	30	2.28
20	4.52	31	4.5	20	2.13	31	2.30
21	4.52	32	4.51	21	2.20	32	2.29
22	4.59	33	4.63	22	2.16	33	2.24
23	4.73	34	4.50	23	2.19	34	2.30
24	4.58	35	4.64	24	2.20	35	2.26
25	4.54			25	2.21		

Table S3. Results of curve fitting analysis for Cu *K*-edge EXAFS spectra of intermediates during the synthesis of CIS−In(Ac)$_3$ QDs.

**Reaction stage**	**Amplitude reduction factor (S_0^2)**	**Bond length (R, Å)**	**Reduced χ^2**	**R-factor**
120 °C	0.648 ± 0.027	2.278 ± 0.007	212.31	0.013
150 °C	0.678 ± 0.029	2.285 ± 0.008	158.45	0.014
180 °C	0.659 ± 0.025	2.291 ± 0.007	172.92	0.011
180 °C 10 min	0.632 ± 0.022	2.303 ± 0.006	115.61	0.010

Table S4. Results of curve fitting analysis for In *K*-edge EXAFS spectra of intermediates during the synthesis of CIS−In(Ac)$_3$ QDs.

**Reaction stage**	**Amplitude reduction factor (S_0^2)**	**Bond length (R, Å)**	**Reduced χ^2**	**R-factor**
120 °C	1.000 ± 0.037	2.450 ± 0.006	13.07	0.011
150 °C	0.996 ± 0.031	2.452 ± 0.005	23.11	0.008
180 °C	0.980 ± 0.041	2.456 ± 0.007	36.39	0.014
180 °C 10 min	1.072 ± 0.034	2.458 ± 0.005	13.05	0.008

Table S5. Results of curve fitting analysis for Cu *K*-edge EXAFS spectra of intermediates during the synthesis of CIS−InI_3 QDs.

**Reaction stage**	**Amplitude reduction factor (S_0^2)**	**Bond length (R, Å)**	**Reduced χ^2**	**R-factor**
120 °C	0.612 ± 0.058	2.291 ± 0.004	117.20	0.011
150 °C	0.634 ± 0.063	2.291 ± 0.009	90.09	0.013
180 °C	0.605 ± 0.031	2.297 ± 0.010	199.24	0.014
180 °C 10 min	0.631 ± 0.021	2.298 ± 0.003	88.46	0.007

Table S6. Summary of the trap density and carrier mobility of electron- and hole-only devices with CIS QDs.

**Device structure**	**Electron-only device (ITO/CIS QDs/ZnO/Ag)**			**Hole-only device (ITO/PEDOT:PSS/CIS QDs/Ag)**		
**Samples**	**V_{TFL} (V)**	**n_{trap} (cm^{-3})**	**μ_e (cm^2 V^{-1}s^{-1})**	**V_{TFL} (V)**	**n_{trap} (cm^{-3})**	**μ_h (cm^2 V^{-1}s^{-1})**
CIS–InI_3 QDs	0.37	1.44×10^{18}	1.37×10^{-4}	0.27	1.05×10^{18}	7.88×10^{-5}
CIS–$In(Ac)_3$ QDs	0.54	3.09×10^{18}	9.44×10^{-5}	0.30	1.71×10^{18}	5.99×10^{-5}

Table S7. PEC hydrogen generation performance comparison of CIS and $CuInSe_2$ (CISe) QD-based photoanodes. For comparison, representative works using Cd QDs, and other QD-based PEC systems are presented. In the sample names, the symbols, “@” and “:”, represent the “core@shell QDs” and “dopant:host materials”, respectively.

Photoanode materials	Electrolyte	Photocurrent density (mA cm^{-2})	Ref.
TiO_2/CIS QDs/ZnS	0.35 M Na_2SO_3 + 0.25 M Na_2S	11.3	This work
TiO_2/CIS QDs/ZnS/SiO_2	0.35 M Na_2SO_3 + 0.25 M Na_2S	8.3	This work
TiO_2/CIS QDs	0.35 M Na_2SO_3 + 0.24 M Na_2S	1.87	S5
$BiVO_4$/Zn:CIS QDs	0.5 M Potassium borate	3.8	S6
TiO_2/MnCIS@ZnS QDs/ZnS	N/A	5.7	S7
TiO_2/CISe QDs/ZnS	0.35 M Na_2SO_3 + 0.25 M Na_2S	10.7	S2
TiO_2/$CuInSe_xS_{2-x}$@ZnS QDs/ZnS	0.35 M Na_2SO_3 + 0.25 M Na_2S	5.3	S8
TiO_2/CISe@CIS QDs/ZnS	0.35 M Na_2SO_3 + 0.25 M Na_2S	3.1	S9
TiO_2/CISe@$CuInSe_xS_{2-x}$@CIS QDs/ZnS	0.35 M Na_2SO_3 + 0.25 M Na_2S	4.5	S10
TiO_2/$CuZnInS_3$ QDs/ZnS	0.35 M Na_2SO_3 + 0.25 M Na_2S	4.4	S11
TiO_2/Cu:ZnInSe QDs/ZnS	0.35 M Na_2SO_3 + 0.25 M Na_2S	11.23	S12
TiO_2/$MnAgInS_2$@Cu:ZnS QDs/ZnS	0.35 M Na_2SO_3 + 0.25 M Na_2S	6.4	S13
TiO_2/Cu:AgInSe@Cu:ZnSe QDs/ZnS	0.35 M Na_2SO_3 + 0.25 M Na_2S	9.1	S14
TiO_2/CdSe@Cd_xZn_{1-x}Se QDs/ZnS	0.35 M Na_2SO_3 + 0.25 M Na_2S	25.4	S15

Table S8. Flat band potential (V_{FB}) and donor concentration (N_d) of CIS QD-sensitized TiO_2 photoanodes measured from Mott–Schottky plots.

Samples	V_{FB} (V_{RHE})	N_d (cm^{-3})
TiO_2/CIS–InI_3/ZnS/SiO_2	–0.055	9.29×10^{20}
TiO_2/CIS–In(Ac)$_3$/ZnS/SiO_2	–0.048	8.66×10^{20}
TiO_2/ZnS/SiO_2	–0.039	1.18×10^{20}
Bare TiO_2	–0.031	1.03×10^{20}

Table S9. Solution resistance (R_s) and charge transfer resistance (R_{ct}) measured by EIS Nyquist plots under 1.0 sun illumination conditions.

Samples	R_s (Ω cm^{-2})	R_{ct} (Ω cm^{-2})
TiO_2/CIS–InI_3/ZnS/SiO_2	3.42	330
TiO_2/CIS–In(Ac)$_3$/ZnS/SiO_2	2.87	888
TiO_2/ZnS/SiO_2	2.72	8,391
Bare TiO_2	2.94	14,613

4. References for the Supporting Information

[S1] M. V. Yakushev, R. W. Martin, A. V. Mudryi, A. V. Ivaniukovich, *Appl. Phys. Lett.* **2008**, *92*, 111908.

[S2] S. Li, S.-M. Jung, W. Chung, J.-W. Seo, H. Kim, S. I. Park, H. C. Lee, J. S. Han, S. B. Ha, I. Y. Kim, S.-I. In, J.-Y. Kim, J. Yang, *Carbon Energy* **2023**, *5*, e384.

[S3] H. Kim, A. Choe, S. B. Ha, G. M. Narejo, S. W. Koo, J. S. Han, W. Chung, J.-Y. Kim, J. Yang, S.-I. In, *ChemSusChem* **2023**, *16*, e202201925.

[S4] H. Kim, J. W. Seo, W. Chung, G. M. Narejo, S. W. Koo, J. S. Han, J. Yang, J.-Y. Kim, S.-I. In, *ChemSusChem* **2023**, *16*, e202202017.

[S5] T.-L. Li, H. Teng, *J. Mater. Chem.* **2010**, *20*, 3656.

[S6] M. Cai, X. Tong, H. Zhao, X. Li, Y. You, R. Wang, L. Xia, N. Zhou, L. Wang, Z. M. Wang, *Small* **2022**, *18*, 2204495.

[S7] R. Wang, X. Tong, A. I. Channa, Q. Zeng, J. Sun, C. Liu, X. Li, J. Xu, F. Lin, G. S. Selopal, F. Rosei, Y. Zhang, J. Wu, H. Zhao, A. Vomiero, X. Sun, Z. M. Wang, *J. Mater. Chem. A* **2020**, *8*, 10736.

[S8] X. Tong, Y. Zhou, L. Jin, K. Basu, R. Adhikari, G. S. Selopal, X. Tong, H. Zhao, S. Sun, A. Vomiero, Z. M. Wang, F. Rosei, *Nano Energy* **2017**, *31*, 441.

[S9] X. Tong, X.-T. Kong, Y. Zhou, F. Navarro-Pardo, G. S. Selopal, S. Sun, A. O. Govorov, H. Zhao, Z. M. Wang, F. Rosei, *Adv. Energy Mater.* **2018**, *8*, 1701432.

[S10] F. Li, M. Zhang, D. Benetti, L. Shi, L. V. Besteiro, H. Zhang, J. Liu, G. S. Selopal, S. Sun, Z. Wang, Q. Wei, F. Rosei, *Appl. Catal. B* **2021**, *280*, 119402.

[S11] C. Liu, X. Tong, A. I. Channa, X. Li, Z. Long, H. Feng, Y. You, R. Wang, F. Lin, C. F. Dee, A. Vomiero, Z. M. Wang, *J. Mater. Chem. A* **2021**, *9*, 5825.

[S12] B. Luo, J. Liu, H. Guo, X. Liu, R. Song, K. Shen, Z. M. Wang, D. Jing, G. S. Selopal, F. Rosei, *Nano Energy* **2021**, *88*, 106220.

[S13] Xia, X. Tong, X. Li, A. Imran Channa, Y. You, Z. Long, A. Vomiero, Z. M. Wang, *Chem. Eng. J.* **2022**, *442*, 136214.

[S14] L. Xia, X. Tong, Y. Yao, Z. Long, M. Cai, L. Jin, A. Vomiero, Z. M. Wang, *Nano Energy* **2024**, *122*, 109302.

[S15] K. Wang, C. Wang, Y. Tao, Z. Tang, D. Benetti, F. Vidal, Y. Liu, M. H. Rummeli, H. Zhao, F. Rosei, X. Sun, *Adv. Funct. Mater.* **2024**, *34,* 2400580.
